# The vimentin rod domain blocks P-selectin-P-selectin glycoprotein ligand 1 interactions to attenuate leukocyte adhesion to inflamed endothelium

**DOI:** 10.1371/journal.pone.0240164

**Published:** 2020-10-13

**Authors:** Fong Wilson Lam, Cameron August Brown, Christian Valladolid, Dabel Cynthia Emebo, Timothy Gerald Palzkill, Miguel Angel Cruz

**Affiliations:** 1 Department of Pediatrics, Baylor College of Medicine, Houston, Texas, United States of America; 2 Center for Translational Research on Inflammatory Diseases, Michael E. DeBakey Veterans Affairs Medical Center, Houston, Texas, United States of America; 3 Department of Pharmacology and Chemical Biology, Baylor College of Medicine, Houston, Texas, United States of America; 4 Department of Medicine, Baylor College of Medicine, Houston, Texas, United States of America; Medical College of Georgia at Augusta University, UNITED STATES

## Abstract

Acute inflammation begins with leukocyte P-selectin glycoprotein ligand-1 (PSGL-1) binding to P-selectin on inflamed endothelium and platelets. In pathologic conditions, this process may contribute to secondary organ damage, like sepsis-induced liver injury. Therefore, developing novel therapies to attenuate inflammation may be beneficial. We previously reported that recombinant human vimentin (rhVim) binds P-selectin to block leukocyte adhesion to endothelium and platelets. In this study, we used SPOT-peptide arrays to identify the rod domain as the active region within rhVim that interacts with P-selectin. Indeed, recombinant human rod domain of vimentin (rhRod) binds to P-selectin with high affinity, with *in silico* modeling suggesting that rhRod binds P-selectin at or near the PSGL-1 binding site. Using bio-layer interferometry, rhRod decreases PSGL-1 binding to immobilized P-selectin, corroborating the *in silico* data. Under parallel-plate flow, rhRod blocks leukocyte adhesion to fibrin(ogen)-captured platelets, P-selectin/Fc-coated channels, and IL-1β/IL-4-co-stimulated human umbilical vein endothelial cells. Finally, using intravital microscopy in endotoxemic C57Bl/6 mice, rhRod co-localizes with P-selectin in the hepatic sinusoids and decreases neutrophil adhesion to hepatic sinusoids. These data suggest a potential role for rhRod in attenuating inflammation through directly blocking P-selectin-PSGL-1 interactions.

## Introduction

Leukocyte (WBC) capture on both platelets and vascular endothelium plays a key role in the initiation of acute inflammation. This early process is mediated through the interactions between adhesion molecules found on WBC, namely P-selectin glycoprotein ligand-1 (PSGL-1) and the β_2_-integrins (CD11a/CD18 [LFA-1] and CD11b/CD18 [Mac-1]) to those found on platelets and endothelial cells (e.g., P-selectin, GP1bα, E-selectin, and ICAM-1) [1–6]. These interactions lead to WBC crawling and eventual transendothelial migration. Normally, inflammation is important in the maintenance of health, such as in infection and wound healing. However, inflammation can also be pathologic, leading to tissue injury and organ dysfunction in both directly affected as well as distant sites, such as secondary acute lung injury [7, 8] and sepsis-associated liver injury [9, 10].

Vimentin is a type 3 intermediate filament protein that normally resides in the cytoplasm of cells to form part of the cytoskeleton and participate in intracellular transport [11]. Recently, vimentin has been identified on the extracellular surface of platelets and endothelial cells [12] in addition to being secreted into the circulation [13]. Although the effect of native circulating vimentin is unclear, we published that exogenous recombinant human vimentin (rhVim) decreases human leukocyte adhesion to platelets and endothelial cells *in vitro* and reduces lung inflammation in endotoxemic mice. This reduction in leukocyte adhesion is through rhVim binding specifically to P-selectin [14]. However, the binding regions on both P-selectin and rhVim to produce those observations were unknown. Additionally, whether rhVim bound to endothelial P-selectin in animals to block leukocyte adhesion was also unknown. In this report, we sought to identify the primary binding domains between rhVim and P-selectin and to test whether shorter fragments of recombinant vimentin exhibited similar effects. The overall aim of this work is to develop small peptides that may serve to selectively attenuate leukocyte adhesion and minimize inflammation. As demonstrated by our data, the rod domain of recombinant vimentin binds specifically to P-selectin to block P-selectin-PSGL-1 interactions. Additionally, the rod domain of vimentin attenuates leukocyte adhesion to platelets and endothelium *in vitro*. Finally, the rod domain of vimentin binds to endothelial P-selectin in liver sinusoids to block neutrophil adhesion in endotoxemic mice.

## Materials and methods

### Ethics statement

Human and animal subjects research was approved by the Institutional Review Boards and Institutional Animal Care and Use Committees, respectively, at Baylor College of Medicine and Michael E. DeBakey Veterans Affairs Medical Center.

### Materials

*Escherichia coli* (Strain M15) was purchased from Qiagen (Germantown, MD). Horseradish peroxidase (HRP)-conjugated anti-polyhistidine antibody, protease inhibitor, bovine serum albumin (BSA), tetramethylbenzidine, mepacrine, fluorescein isothiocyanate (FITC)-dextran (150 kDa), Atto 550 NHS ester, and endotoxin (L3024) were purchased from Sigma-Aldrich (St. Louis, MO). Dulbecco’s phosphate buffered saline without calcium or magnesium (DPBS) and with calcium and magnesium (DPBS^+/+^) was purchased from Gibco (Thermo Fisher Scientific; Waltham, MA). Recombinant human interleukin-1β (rhIL-1β), recombinant human interleukin-4 (rhIL-4), P-selectin/Fc (P-sel/Fc), and P-selectin glycoprotein ligand-1/Fc (PSGL-1/Fc) were purchased from R & D Systems (Minneapolis, MN). Human umbilical vein endothelial cells (HUVEC) and HUVEC media were purchased from Lonza (Morristown, NJ). Fibronectin was purchased from Advance Biomatrix (Carlsbad, CA). Antibodies for intravital microscopy were as follows: BV421-anti-Ly6G antibody (clone 1A8; BioLegend; San Diego, CA), PerCP-eFluor 710-anti-P-selectin antibody (clone Psel.KO2.3; Invitrogen; Thermo Fisher Scientific), and DyLight 488-anti-platelet antibody (X488; emfret analytics; Eibelstadt, Germany). BioFlux microfluidic plates were purchased from Fluxion Biosciences (Alameda, CA). Amine reactive 2^nd^ generation sensors were purchased from Pall ForteBio LLC (Bohemia, NY). Derivatised cellulose membranes for SPOT peptide arrays were purchased from Intavis (CEM Corporation; Matthews, NC). IR800CW dye and Tris-buffered saline (TBS) Blocking buffer were purchased from Li-Cor (Lincoln, NE).

### SPOT peptide array

SPOT peptide arrays were utilized to determine the potential interaction sites between recombinant vimentin and P-selectin. The peptide arrays were synthesized on a derivatized cellulose membrane using the MultiPep RS automated peptide synthesizer (Intavis, Köln, Germany) [15]. Full-length sequences of vimentin (SPOT-Vim; NCBI reference sequence NP_003371.1) and P-selectin (SPOT-Psel; UniProt P16109.3) were used on separate membranes. Peptides of 20 amino acids (aa) in length with frame shifts of 2 aa (vimentin) or 3 aa (P-selectin) were placed per spot (S1 and S2 Tables). Once completed, the membranes were washed in methanol for 10 minutes followed by TBS (50 mM Tris, 140 mM NaCl; pH 7.5) containing 0.05% Tween-20 and 0.1% BSA. The membranes were blocked using TBS blocking buffer for 4 hours at room temperature. P-sel/Fc or rhRod was labeled with IR800CW dye per the manufacturer’s recommendations and then dialyzed against DPBS to detect protein binding sites on the SPOT membranes. Membranes were then incubated with 1 μg/mL P-sel/Fc-IR800 (on SPOT-Vim) or rhRod-IR800 (on SPOT-Psel) for 1 hour at room temperature. All membranes were washed in TBS+0.05% Tween-20 and 0.1% BSA, and then imaged on a Li-Cor Odyssey detector using the 800 nm channel for 5 minutes.

### *In silico* protein interaction

The protein-protein interaction between P-selectin and the rod domain of vimentin was simulated using the HADDOCK2.2 docking server prediction interface with default parameters [16]. Residues of P-selectin and the rod domain of vimentin that were shown to be important for binding by SPOT synthesis were used to guide the interaction between the two proteins. P-selectin residues 1–24, 38–75, 107–129 were selected as active residues for docking using PDB 1G1Q, while residues 114–138 (PDB: 1GK7) and 280–295 (PDB: 3TRT) were selected for vimentin. All PDBs were stripped of hetero atoms prior to docking. Because the rod domain required two different structures, two separate docking runs were performed. The top model from each run was considered for further analysis (S1 Methods). The models are shown using Chimera software (1.13; UCSF).

### Generation of full-length (rhVim) and the rod domain (rhRod) of recombinant vimentin

**Fig 1:**
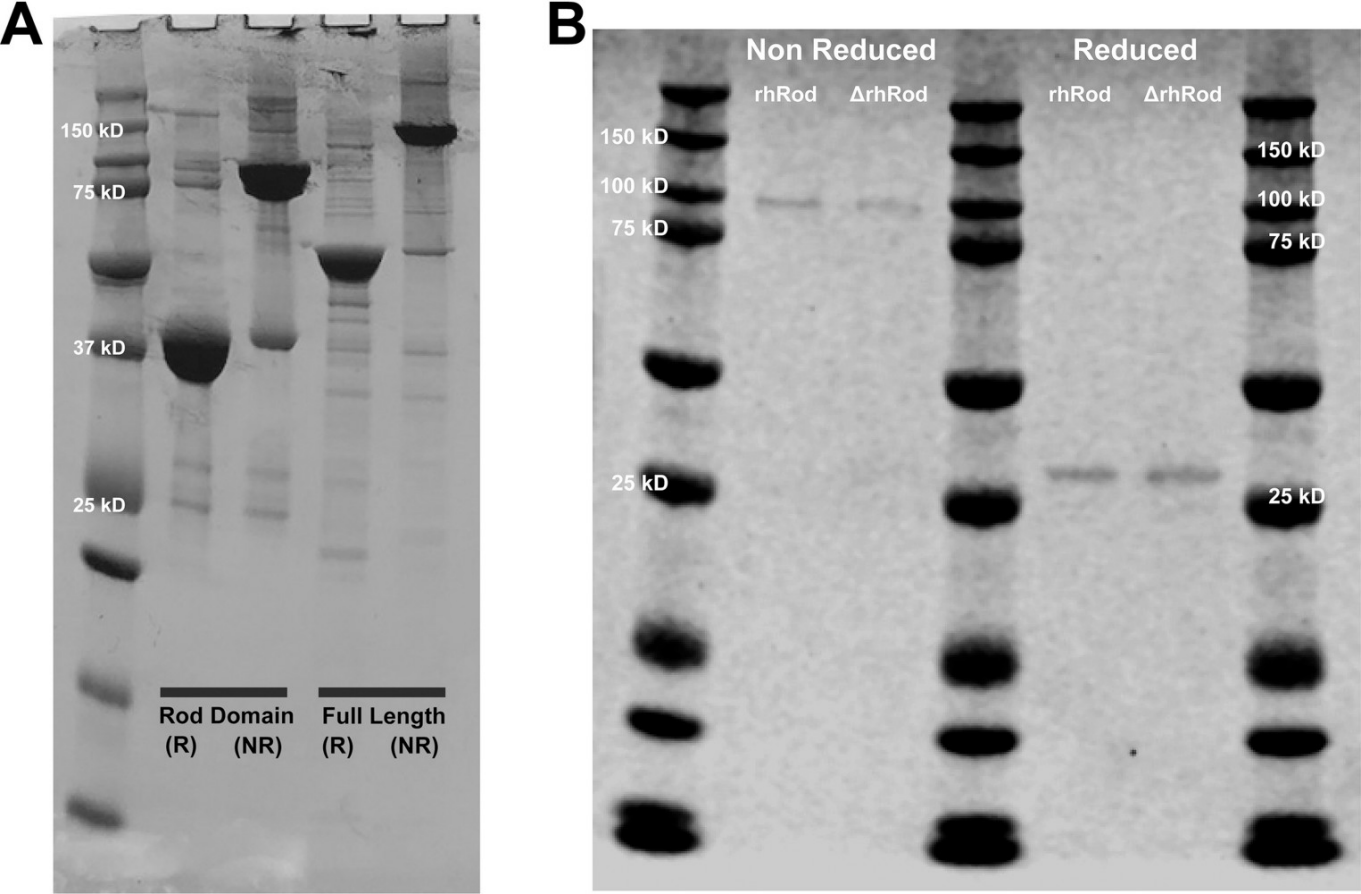
Coomassie stain of (A) rhRod and rhVim in reducing (R) and non-reducing (NR) conditions and (B) rhRod and ΔrhRod in non-reducing and reducing conditions. (A) Non-reduced rhVim and rhRod are approximately twice the size of reduced protein, suggesting dimerization. (B) Coomassie stain of rhRod and ΔrhRod are similar in both the non-reduced and reduced states.

Protein expression and purification were performed as before [14]. The sequence NM_00380.3 (NCBI) was used to synthesize both full-length human vimentin (base pairs 418–1,814) and the rhRod (base pairs 717–1,652; aa residues 96–407). The sequences for either rhVim or rhRod were inserted into pQE-30 vector (GenScript). The resultant plasmids were transformed into *Escherichia coli (*strain M15). *E*. *coli* harboring rhRod or rhVim pQE-30 were grown to mid-log phase at 37°C and protein expression was induced using 1.5 mM isopropyl β-D-1-thiogalactopyranoside (IPTG) for 4 hours. The *E*. *coli* bacteria were pelleted and lysed to obtain inclusion bodies which were then lysed with lysis buffer (6 M guanidine hydrochloride, 25 mM Tris, pH 7.4), supplemented with 1x protease inhibitor at room temperature for 2 h with constant stirring. Cell lysates were centrifuged at 19,000 rpm for 30 minutes and the supernatants filtered using a 0.45 μm filter before loading onto the Ni^2+^ affinity column previously equilibrated with binding buffer (7 M urea, 25mM Tris, pH 8). Bound proteins were eluted with elution buffer (200 mM imidazole, 8 M urea, 25 mM Tris, pH 8). The purified sample was serially dialyzed against decreasing concentrations of urea in Tris buffer at 4°C. Samples were ultimately dialyzed against 20 mM sodium phosphate buffer, pH 8, overnight at 4°C and then filtered using a 0.22 μm filter under sterile conditions. Absorbance was measured at 280 nm to determine protein concentration using extinction coefficients and molecular weights of 21,425 L/mol/cm and 57 kDa for rhVim or 15,350 L/mol/cm and 38.2 kDa for rhRod. Purity was confirmed using SDS-PAGE and Coomassie blue stain. Like full-length vimentin, the rhRod also dimerized in non-reducing conditions (Fig 1A).

### Bio-layer interferometry

We used bio-layer interferometry (BLI; Octet Red 384; Pall ForteBio LLC) to determine the K_D_ of P-selectin and PSGL-1 to rhRod. We immobilized rhRod 50 μg/mL onto amine reactive 2nd generation sensors (AR2G; Pall ForteBio LLC) using 10 mM Sulfo-*N*-hydroxysuccinimide and 20 mM 1-ethyl-3-(3dimethylaminopropyl)-carbodiimide. The amine coupling reaction was quenched with 1 M ethanolamine-HCl, pH 8.5, and then washed with DPBS. rhRod-immobilized sensors were then used to assess binding kinetics to P-sel/Fc and PSGL-1/Fc at concentrations ranging from 0–1,000 nM. In a separate series of experiments, P-sel/Fc or PSGL-1/Fc (10 μg/mL) were immobilized on Anti-human IgG Fc sensors (AHC; Pall ForteBio, LLC) in DPBS. The P-sel/Fc or PSGL-1/Fc immobilized sensors were then used to assess the binding kinetics to rhVim or rhRod at concentrations ranging from 0–1,000 nM.

To assess whether the tertiary structure of rhRod was important in its interaction with P-selectin, we performed additional experiments in which rhRod (1,000 nM) was split into room temperature and heat inactivation aliquots. rhRod was placed into boiling water (100°C) for 10 minutes to make heat denatured rhRod (ΔrhRod). Both rhRod and ΔrhRod were kept at room temperature until ΔrhRod reached room temperature. There was no change in protein concentration between rhRod and ΔrhRod. SDS-PAGE followed by Coomassie blue stain was performed on the rhRod (1,000 nM) and ΔrhRod (1,000 nM) in both non-reduced (Laemmli buffer only) and reduced (Laemmli buffer with 2-mercaptoethanol followed by boiling for 10 minutes) conditions. There was no difference in the Coomassie stain between rhRod and ΔrhRod in either the reduced and non-reduced states (Fig 1B). Binding of rhRod and ΔrhRod to immobilized P-sel/Fc was performed as above.

To determine if rhRod blocked PSGL-1 binding to P-selectin, we performed a separate set of experiments, in duplicate. We first performed K_D_ experiments with immobilized P-sel/Fc against PSGL-1/Fc to determine which PSGL-1/Fc concentration to use. P-sel/Fc was loaded onto AR2G sensors as above. The P-sel/Fc-immobilized sensors were then placed into wells containing recombinant human PSGL-1/Fc (Sino Biological Inc.) at concentrations ranging from 0–1,000 nM to assess the K_D_. Once the K_D_ for PSGL-1/Fc was determined, we proceeded to the rhRod blocking experiments. P-sel/Fc was loaded onto AR2G sensors, followed by placement into wells containing rhRod at concentrations ranging from 0–1,000 nM for association followed by dissociation in DPBS. Then, the sensors were placed into wells containing 500 nM PSGL-1/Fc (Sino Biological Inc.) to determine if increasing concentrations of rhRod inhibited PSGL-1/Fc binding. The PSGL-1/Fc response was then plotted against the rhRod concentration and analyzed using non-linear regression to determine the rhRod IC50.

All buffers used for association and dissociation kinetics were DPBS. Regeneration was performed using 10 mM glycine, pH 1.7–1.75. Data were analyzed using Octet System Data Analysis software (Pall ForteBio LLC).

### Leukocyte and isolated neutrophil collection

Citrated whole blood was collected from healthy human subjects to study whole blood (3.2% sodium citrate) or isolated neutrophil (iPMN; citrate phosphate dextrose). To evaluate neutrophils, citrated blood was processed as before to isolate neutrophils [1, 14]. Briefly, erythrocytes were sedimented using dextran in sodium chloride. Leukocyte-rich plasma was removed and washed in DPBS and placed atop a Ficoll type 400-diatrizoate sodium cushion for neutrophil separation. Erythrocytes were lysed using water followed by addition of 10X DPBS to stop lysis. Isolated neutrophils were washed in DPBS and resuspended in DPBS^+/+^ with 10 mM glucose. Both whole blood leukocytes and platelets and iPMN were labeled with 10 μM mepacrine for 20 minutes at 37°C to aid in visualization [17].

### HUVEC preparation

HUVEC were prepared and seeded onto Bioflux microfluidic chambers as before [14]. Briefly, P3-P5 HUVEC were cultured in T75 flasks until >90% confluence before passage onto fibronectin (100 μg/mL)-coated channels. When HUVEC reached 100% confluence in the channels, they were co-stimulated with IL-1β (50 U/mL) and IL-4 (20 ng/mL) for 24 hours to stimulate P-selectin upregulation [18].

### Leukocyte and iPMN flow adhesion assays

Parallel-plate flow adhesion studies were performed using BioFlux microfluidic plates as described previously [14, 19]. To prepare the samples for parallel plate flow, mepacrine labeled whole blood or iPMN from individual subjects were aliquoted into separate tubes from the stock source. Then equal volumes of either control, rhRod, or rhVim were added to each tube prior to perfusion. Whole blood or iPMN was perfused over microfluidic channels coated with either fibrinogen (100 μg/mL), P-sel/Fc (10 μg/mL), or IL-1β/IL-4-stimulated HUVEC. In assays measuring whole blood leukocyte adhesion to fibrin(ogen)-bound platelets, whole blood was first perfused over fibrin(ogen)-coated channels at 5 dyn/cm^2^ for 10 minutes to capture platelets. At this shear, there was no leukocyte adhesion detected. The shear stress was then reduced to 2 dyn/cm^2^ to assess for leukocyte adhesion to fibrin(ogen)-captured platelets. In assays measuring iPMN adhesion to either P-sel/Fc- or IL-1β/IL-4-co-stimulated HUVEC, the shear stress was maintained at 2 dyn/cm^2^. All assays were imaged using the FITC channel with 200-ms exposure and either a 5X, 10X, or 20X objective lens (Zeiss). Static and time-lapsed images (100 frames) were captured (S1 Video). Image analysis was performed using FIJI/Image J (NIH) [20]. Quantification of platelet adhesion to fibrin(ogen)-coated surfaces was determined by measuring the mean fluorescence intensity of identically sized regions of interest (1200 x 300 pixels) between control- and rhRod (50 μg/mL)-treated whole blood. Quantification of leukocyte adhesion was performed by counting the leukocytes adhered in a field of view.

### Intravital microscopy of murine liver

To study whether rhRod bound to P-selectin and blocked neutrophil adhesion to liver sinusoids *in vivo*, we labeled rhRod with Atto 550. Briefly, Atto 550 was reconstituted in DMSO and immediately added to rhRod and incubated for 2 hours, per manufacturer instructions. Following incubation, the labeled protein was dialyzed twice against 20 mM sodium phosphate pH 8 using a 7 kDa MWCO Slide-a-Lyzer cassette and then sterilized using a 0.2 μm filter. The dialysate was also sterilized via filtration and used as buffer control. Antibodies used to visualize neutrophils (BV421-anti-Ly6G) and P-selectin (PerCP-eFluor 710-anti-P-selectin) were dialyzed twice against sterile, endotoxin-free DPBS to remove sodium azide for use in animals.

Twelve- to sixteen-week old C57Bl/6 mice of both sexes (20–30 g) were used for liver intravital microscopy. Atto 550-rhRod (3 mg/kg) or control was injected intraperitoneally into mice, followed by an intraperitoneal injection of endotoxin (LPS; from *Escherichia coli* O111:B4) 5 mg/kg. Three and one-half hours after LPS injection, mice were prepared for intravital microscopy as we have previously described [21]. Briefly, mice were anesthetized using pentobarbital and underwent tracheostomy (to facilitate breathing) and internal jugular catheterization (for antibody administration) followed by liver exteriorization as described by Marques *et al*. [22], with modifications. Mice were placed supine on a custom-made stage with the liver overlying a glass coverslip wetted with warmed saline and surrounded with wet saline-soaked gauze. Mice were kept euthermic at 37C using radiant warmers and monitored with a rectal thermometer. Anesthesia was maintained using an isoflurane delivery device (RoVent with SomnoSuite; Kent Scientific) with 1–3% isoflurane delivered. Mice were intravenously injected with FITC/dextran (150 kD; to label the vasculature; 250 μg/mouse), BV421-anti-Ly6G antibody (to label neutrophils; 3 μg/mouse), and PerCP-eFluor 710-anti-P-selectin antibody (4 μg/mouse) for visualization. Early experiments were performed to determine whether the P-selectin detected within the liver sinusoids were due to the accumulation of platelets. In these experiments, mice were anesthetized and instrumented as above (without endotoxin exposure) and injected with the following antibodies intravenously: DyLight 488-anti-GPIbβ (X488; 6 μg/mouse; emfret analytics), BV421-anti-Ly6G, PerCP-eFluor 710-anti-P-selectin, and Texas Red-Dextran (150 kD; 250 μg/mouse; Sigma-Aldrich). All animals were euthanized under a surgical plane of anesthesia at the end of the experiments.

### Imaging and image analysis of intravital microscopy experiments

Mice were imaged on an Olympus FV3000RS laser scanning confocal microscope at 30 fps using a 60X/NA1.30 silicone oil objective with 1X optical zoom using the resonance scanner. This allows for simultaneous excitation and detection of up to four wavelengths. Ten 1-minute fields of view were analyzed per mouse using FIJI/ImageJ software. Background noise was removed using a median filter (1 pixel) prior to image analysis. Vascular area was measured in each field using the selection brush in the FITC (dextran) channel. Firmly adherent neutrophils were defined as neutrophils moving less than 1 cell body over the course of 1 minute (S1 Fig; S2 Video). The number of neutrophils per mm^2^ vessel area was then determined for each animal. To estimate the blood flow velocity within the sinusoids, red blood cells (RBC) were identified using negative staining within the dextran channel [23] and their velocities were measured using MTrackJ plugin in FIJI/ImageJ [24]. Tracks were created by manually selecting the center of RBC through a series of frames. An average of 10 tracks in different vessel segments were created per field of view (S2 Fig). The mean velocities were calculated in the MTrackJ plugin using a frame interval of 33.3 ms and then analyzed to compare the control- and rhRod-treated groups.

### Histologic imaging of vimentin expression in endotoxemic mice

To determine whether liver vimentin expression was increased 4 hours after endotoxin exposure, 14- to 19-week old C57Bl/6 mice of both sexes were used for histology. Mice were given either an intraperitoneal injection of endotoxin (LPS; from *Escherichia coli* O111:B4) 5 mg/kg or normal saline. Four hours later, mice were euthanized by blinded investigators and livers fixed in 10% buffered formalin and then processed for histology. Sections were treated with Peroxidazed 1 (Biocare Medical) prior to heat induced epitope retrieval using Rodent Decloaker (Biocare Medical). Background Punisher (Biocare Medical) was used to reduce non-specific binding prior to primary antibody incubation with rabbit anti-vimentin antibody (Bioss USA). Detection was performed using Rabbit-on-Rodent HRP-Polymer (Biocare Medical) and 3,3’-diaminobenzidine (DAB) administration. Stained sections were imaged using light microscopy on an Olympus IX83 light microscope using a 40X/NA1.25 silicone oil objective. Five 40X fields of view were imaged per section using identical light intensity and exposure times. Images were uploaded into FIJI/ImageJ and processed similar to previously reported [25]. Images first underwent colour deconvolution (version 1.7) using the H DAB vector to identify the DAB channel [26]. A Gaussian filter (radius of 4 pixels) was applied to reduce background noise. Finally, the automated triangle threshold was used to determine the percent area of DAB staining [27] (See S3 Fig for an example).

### Statistical analysis

K_D_ values were calculated using ForteBio Data Analysis software. Additional data analysis was performed using Prism 8 (GraphPad Software Inc). To determine the IC50 of rhRod in blocking PSGL-1 binding to P-selectin, a nonlinear regression was performed. For human studies, data were analyzed using either Student’s paired t-tests (two groups) or repeated measures analysis of variance (ANOVA) with Dunnett’s multiple comparison test (three or more groups) to compare treatments to controls within each subject. For murine studies, data were analyzed using Student’s unpaired t-test. Data are represented as mean ± SEM. P-values <0.05 (*), <0.01 (**), and <0.005 (***) were considered significant. K_D_ values in which R^2^ <0.8 were considered indeterminate.

## Results

### Vimentin binds to P-selectin mainly through the rod domain

**Fig 2:**
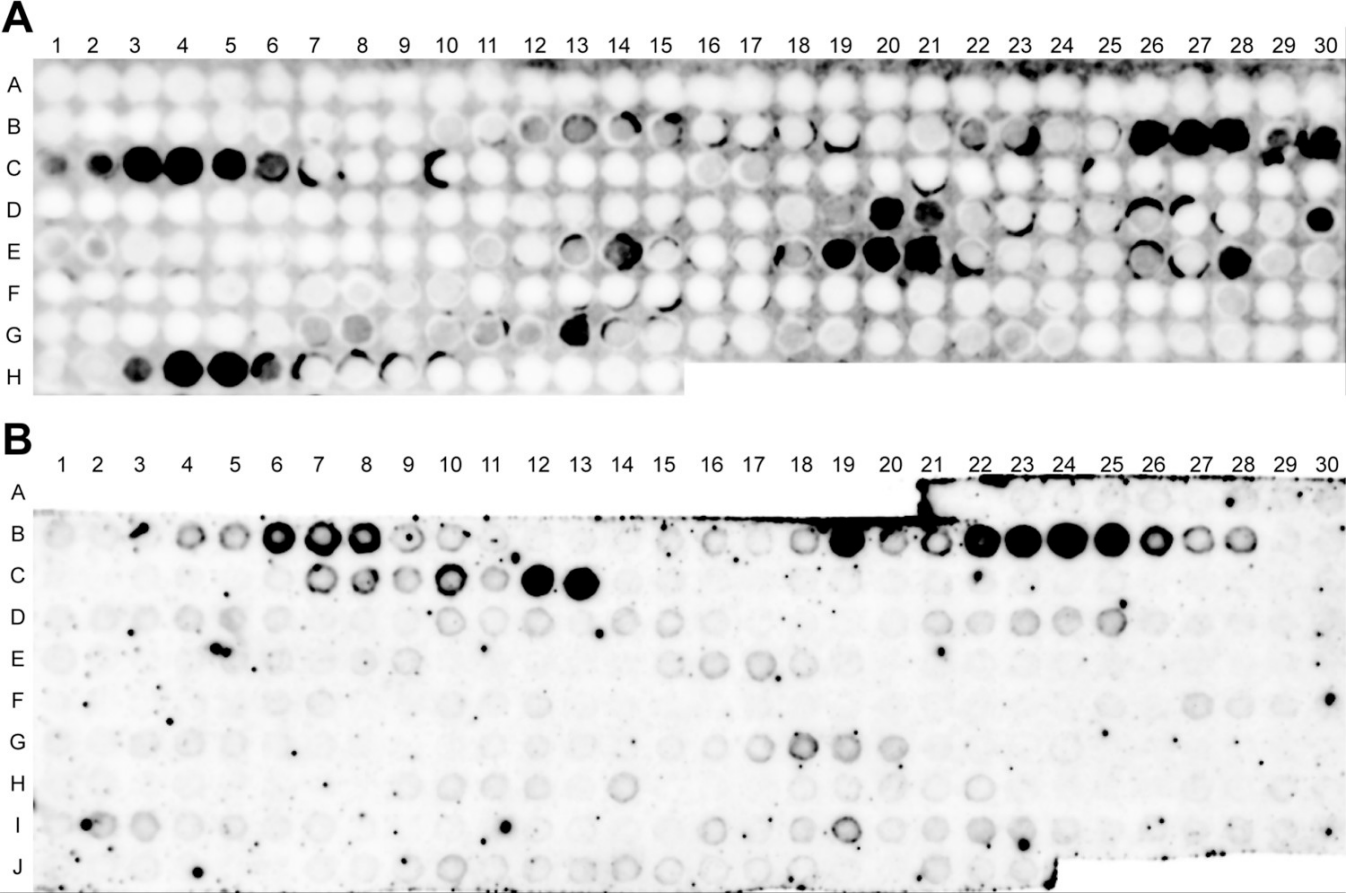
SPOT-peptide membranes of (A) full-length human vimentin and (B) human P-selectin. The dark spots indicate locations in which IR800-labeled proteins bound to 20 amino acid residues from (A) full-length vimentin or (B) P-selectin. (A) IR-labeled P-sel/Fc bound to 20 amino acid spots of full-length vimentin that are located primarily in the rod domain. There is an 18-aa overlap between adjacent spots. (B) IR800-labled rhRod bound to 20 amino acid spots from P-selectin. There is a 17-aa overlap between adjacent spots. Letters and numbers correlate to the 20-aa sequence list in S1 Table (full-length vimentin) & S2 Table (P-selectin).

**Fig 3:**
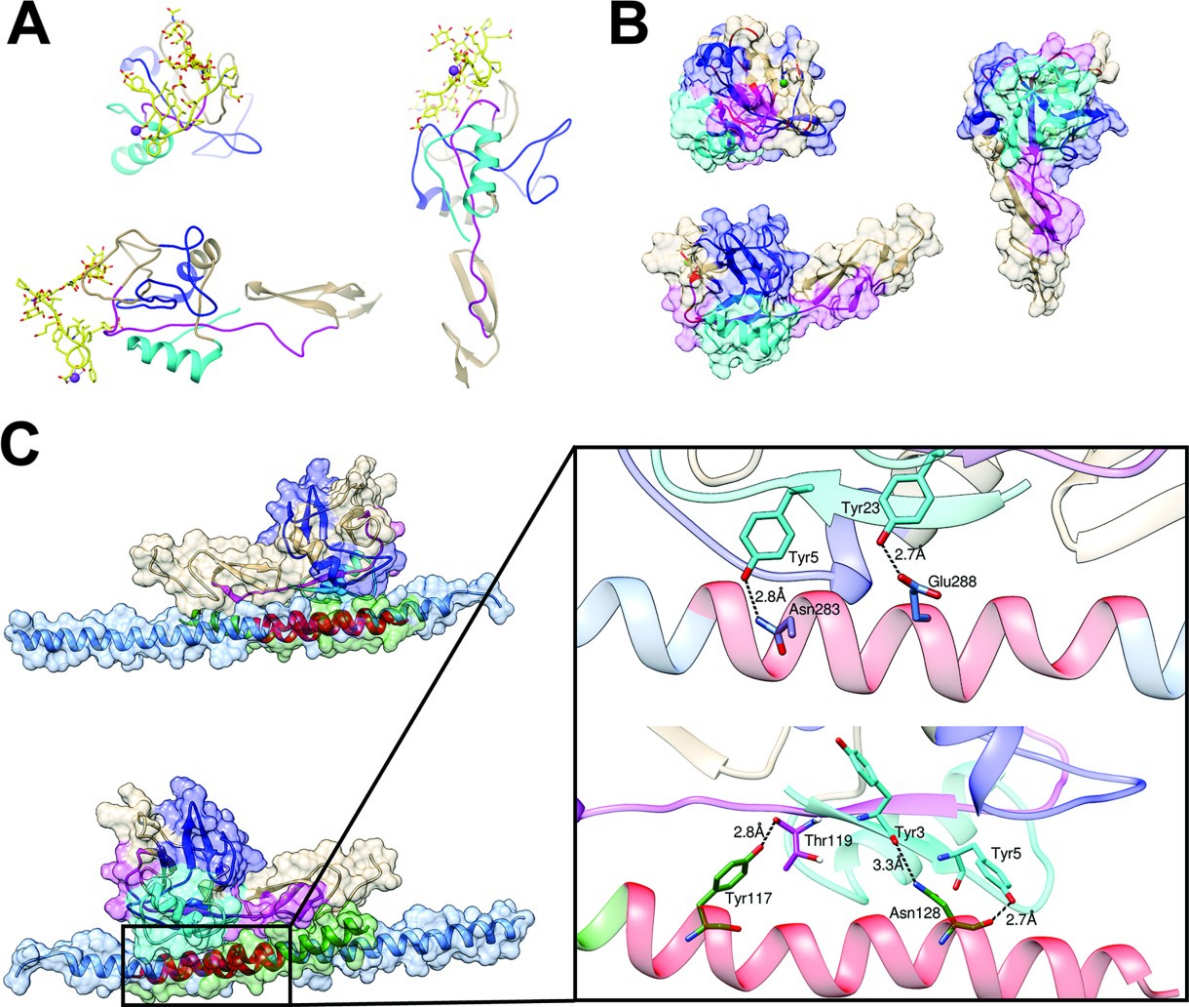
Three views of the crystallized lectin/EGF-domain of P-selectin showing the rhRod-binding regions based on the SPOT array. P-selectin is shown (A) complexed with the binding region of PSGL-1, SGP-3 (yellow; PDB 1G1S) and (B) without SGP-3 (PDB 1G1Q). The rhRod-binding regions are highlighted based on the SPOT array (see S1 Table; cyan: SPOTs #14–16 (aa residues 43–65), blue: SPOTs #27–33 (aa residues 79–116), and magenta: SPOTs #50–51 (aa residues 148–170)). The rhRod binding regions overlap with where PSGL-1/SGP-3 interacts with P-selectin (ribbon regions highlighted in red). (C) Two views of the top *in silico* prediction model of rhRod (green) binding to P-selectin in the region correlating to aa residues 107–129 (Psel SPOT #31–42).

Based on our published data regarding the interaction between rhVim and P-selectin [14], we created a SPOT peptide array of 20-aa residues with 2 aa frame shifts of the full length of vimentin. IR800-labeled P-sel/Fc protein bound to multiple spots, primarily corresponding to the rod domain of vimentin (Fig 2A). To determine the region of P-selectin to which the rod domain binds, we created rhRod in *E*. *coli* (M15 strain). We then performed a separate SPOT peptide array using 20-amino acid residues with a frame shift of 3 aa of P-selectin. Using IR800-labeled rhRod, we found a cluster of binding sites for rhRod (Fig 2B). Using the data from both SPOT membranes and published data on the crystal structure of P-selectin [28], we mapped where rhRod is expected to bind to P-selectin. The major sites identified on the SPOT array correspond to regions related to the PSGL-1 binding region in the lectin and epidermal growth factor (EGF) domain of P-selectin (Fig 3A and 3B).

*In silico* docking using the HADDOCK2.2 server was performed using the residues that were previously shown to play a role in binding via the SPOT peptide array. There are multiple crystal structures for vimentin [11, 29–31]; however, no one structure contained all the active residues that were shown to bind P-selectin. Therefore, multiple docking runs were performed to cover all active residues for vimentin using two different structures from the PDB (1GK7 and 3TRT). The top structures for each docking run were analyzed further. Docking runs were performed using only one helix of the vimentin, which has a coiled coil structure of two identical α-helices. The top structures for both 1GK7 and 3TRT docked with P-selectin showed that the single helix of vimentin binds to EGF-like domain of P-selectin (Fig 3C). Because the docking run was performed without solvent, only direct interactions can be measured between docked vimentin and P-selectin. With both 3TRT and 1GK7, the vimentin rod domain is within hydrogen bond distance of Tyr3 and Tyr5 of P-sel, while 1GK7 makes an additional hydrogen bond to Thr119 of P-sel (Fig 3C, insert). Both models also show that vimentin rod domain extends towards the PSGL-1 binding site of P-selectin, likely introducing significant steric hindrance for the binding of the ligand. Taken together, these data provide mechanistic insight to how recombinant vimentin binds to P-selectin to block leukocyte adhesion, primarily via the rod domain of vimentin.

### rhRod blocks P-selectin-PSGL-1 interactions through binding P-sel/Fc, but not PSGL-1/Fc

**Fig 4:**
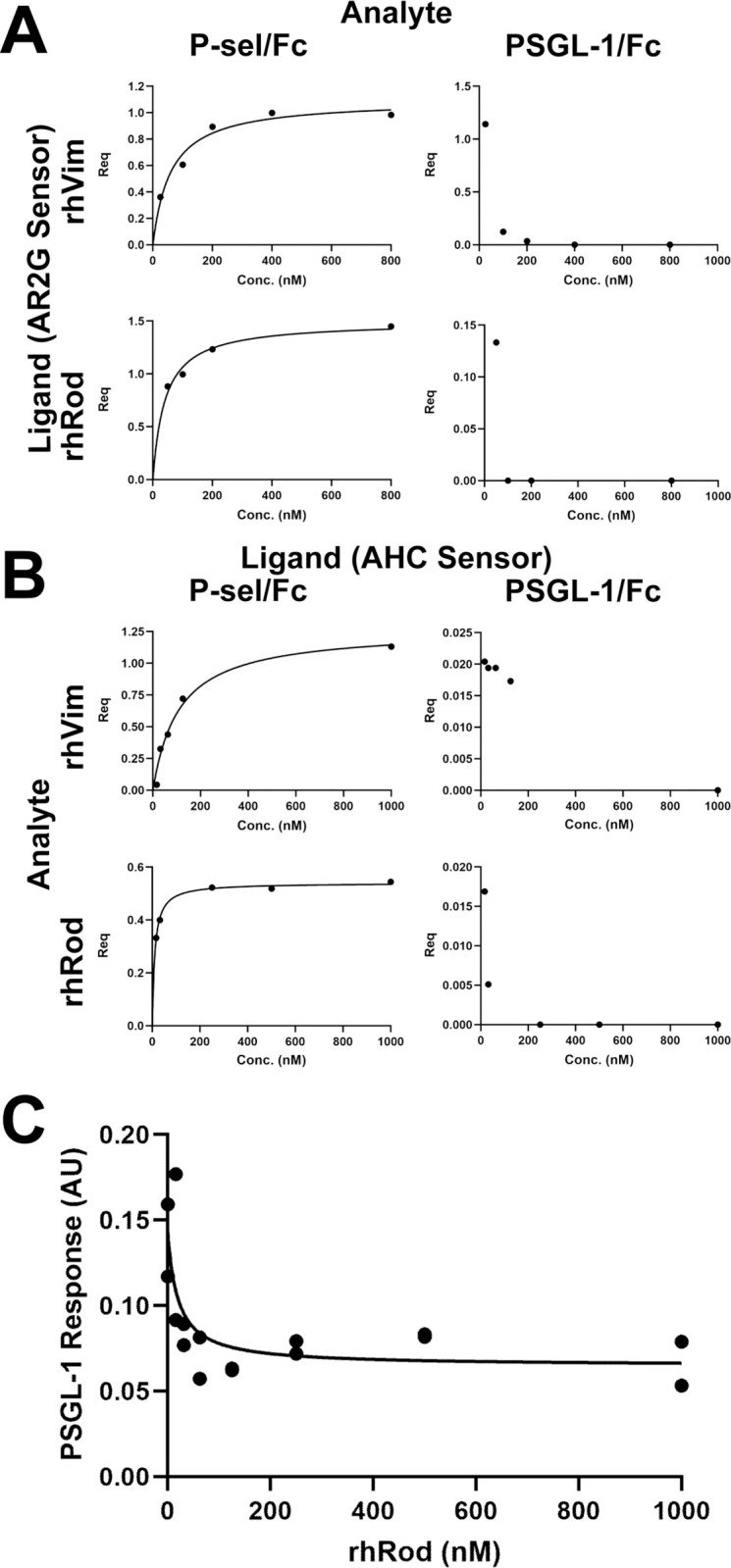
rhRod binds to P-selectin and blocks PSGL-1-P-selectin interactions. (A & B) Steady state binding analysis curves of R_eq_ versus concentration of rhVim or rhRod to either P-sel/Fc or PSGL-1/Fc. rhVim and rhRod both have strong binding to P-sel/Fc regardless of whether the immobilized compound is (A) rhVim or rhRod (on AR2G sensors) or (B) P-sel/Fc or PSGL-1/Fc (on AHC sensors). Neither rhVim nor rhRod binds to PSGL-1/Fc. (C) Increasing concentrations of rhRod blocked PSGL-1/Fc binding to immobilized P-sel/Fc.

We previously published that recombinant human vimentin binds with high affinity to P-selectin, but not PSGL-1 [14]. Given the data presented on the SPOT peptide array, we created a recombinant form of the rod domain of vimentin. We used biolayer interferometry to assess the binding kinetics between rhRod and rhVim to either P-sel/Fc or PSGL-1/Fc. Regardless of whether rhRod and rhVim were the immobilized ligand or the analyte in solution, there was high affinity of both rhRod and rhVim to P-sel/Fc, but not to PSGL-1 (Table 1 and Fig 4A and 4B). These data are congruent with our previous observation [14]. In a separate set of experiments, we tested whether heat inactivation of rhRod would affect its binding to P-sel/Fc. As compared to the baseline rhRod (K_D_ 280 ± 61 nM; R^2^ 0.9754), heat inactivated rhRod did not bind to immobilized P-selectin (K_D_ 7.6x10^9^ M; R^2^ 0).

**Table 1:** K_D_ determination by biolayer interferometry.

Sensor	Ligand	Analyte	K_D_ (nM)	R^2^
AR2G	rhVim	P-sel/Fc	60 ± 12	0.9562
	rhVim	PSGL-1/Fc	ND	0
	rhRod	P-sel/Fc	41 ± 6	0.955
	rhRod	PSGL-1/Fc	ND	0
AHC	P-sel/Fc	rhVim	110 ± 19	0.9739
	P-sel/Fc	rhRod	10 ± 0.6	0.9911
	PSGL-1/Fc	rhVim	ND	0
	PSGL-1/Fc	rhRod	ND	0

AR2G: amine reactive 2^nd^ generation sensor; AHC: anti-human IgG capture sensor; ND: not determined. R^2^<0.8 was determined to be indeterminate.

Based on this information and our *in silico* data (Fig 3 and S1 Methods), we tested whether rhRod blocked PSGL-1/Fc binding to immobilized P-sel/Fc. The K_D_ of PSGL-1/Fc binding to immobilized P-sel/Fc was 240 ± 67 nM (R^2^ 0.9374). In our blocking experiments, we analyzed the PSGL-1/Fc association response after first placing P-sel/Fc-immobilized sensors into decreasing concentrations of rhRod. The IC50 of rhRod to block PSGL-1/Fc binding to P-sel/Fc was 20.03 ± 17.41 nM (95% CI 2.218–116 nM; Fig 4C). These data confirm our SPOT array data that P-selectin interacts with vimentin primarily in the rod domain to block P-selectin-PSGL-1 interactions.

### rhRod blocks leukocyte adhesion to fibrin(ogen)-captured platelets, inflamed HUVEC, and P-sel/Fc-coated plates

**Fig 5:**
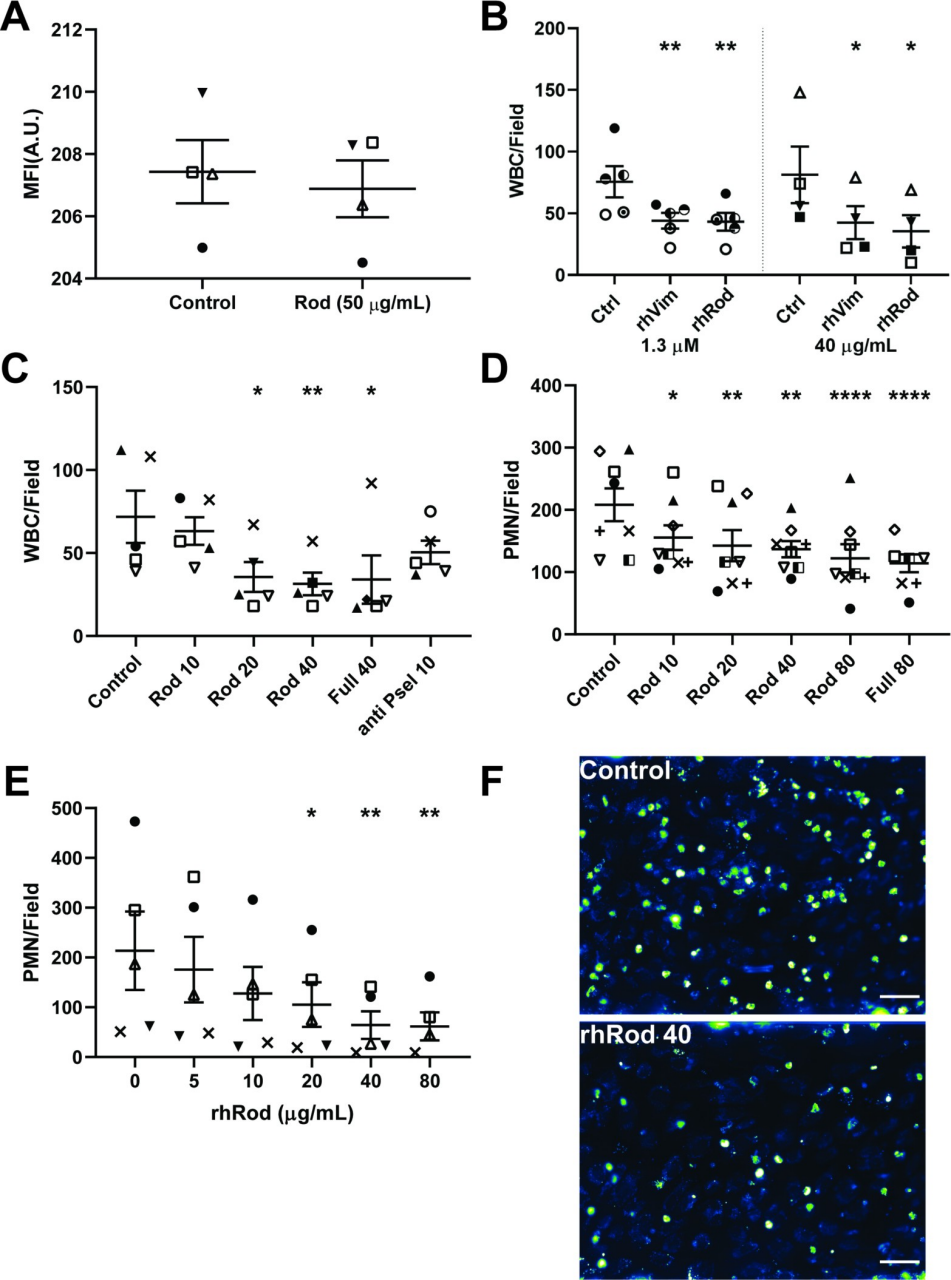
rhRod blocks leukocyte adhesion to platelets, inflamed endothelium, and P-selectin-coated channels. (A) Mepacrine-labeled whole blood perfusing over fibrin(ogen)-coated channels at 5 dyn/cm^2^. rhRod did not affect platelet adhesion to fibrin(ogen)-coated channels, based on mean fluorescence intensity. (B) rhRod decreased leukocyte adhesion to fibrin(ogen)-captured platelets similar to rhVim at equal molar (1.3 μM) and mass (40 μg/mL) concentrations. (C) rhRod decreased leukocyte adhesion to IL1β/IL4-co-stimulated HUVEC. (D) rhRod also decreased isolated PMN adhesion to IL1β/IL4-co-stimulated HUVEC. (E) Finally, rhRod decreased isolated PMN adhesion to P-selectin coated channels in a dose dependent fashion. Leukoocyte adhesion was measured at shear stress of 2 dyn/cm^2^. (F) Representative images of mepacrine-labeled whole blood leukocytes perfused over IL1β/IL4 co-stimulated HUVEC in the presence of control buffer (top) and rhRod 40 μg/mL (bottom). Each subject is represented by a unique symbol. Scale bar = 50 μm. *p<0.05, **p<0.01, ***p<0.005, & ****p<0.001.

We tested whether rhRod blocked leukocyte adhesion to fibrin(ogen)-captured platelets. Whole blood platelets were first captured by fibrin(ogen) at a higher shear stress (5 dyn/cm^2^). There was no effect of rhRod on the density of platelets (measured by mean fluorescence intensity) captured by fibrin(ogen) when perfused at 5 dyn/cm^2^ (Fig 5A). Leukocytes started to adhere after the shear stress was reduced to 2 dyn/cm^2^. Both rhRod and rhVim reduced leukocyte adhesion to fibrinogen-coated channels at equal molar (1.3 μM) and mass (40 μg/mL) concentrations (Fig 5B).

We then tested the ability of rhRod to block leukocyte and isolated neutrophil adhesion to inflamed endothelium. Mepacrine-labeled whole blood was perfused over IL-1β/IL-4 co-stimulated HUVEC in the presence of increasing concentrations of rhRod, rhVim, and anti-P-selectin blocking antibody. rhRod reduced, but did not completely abolish, leukocyte adhesion to inflamed endothelium, similar to full-length vimentin (Fig 5C). rhRod also blocked isolated neutrophil adhesion to inflamed endothelium, albeit to a lesser extent than as seen with whole blood leukocytes (Fig 5D).

Finally, based on our binding assays, we tested whether rhRod blocked iPMN adhesion to P-sel/Fc-coated channels. Indeed, rhRod decreased iPMN adhesion to P-sel/Fc-coated channels in a dose-dependent fashion (Fig 5E). Taken together, these data show that rhRod has similar efficacy as rhVim in blocking leukocyte adhesion to platelets, inflamed endothelium, and P-selectin coated channels.

### rhRod colocalizes with P-selectin in and decreases neutrophil adhesion to liver sinusoids after endotoxin exposure in mice

**Fig 6:**
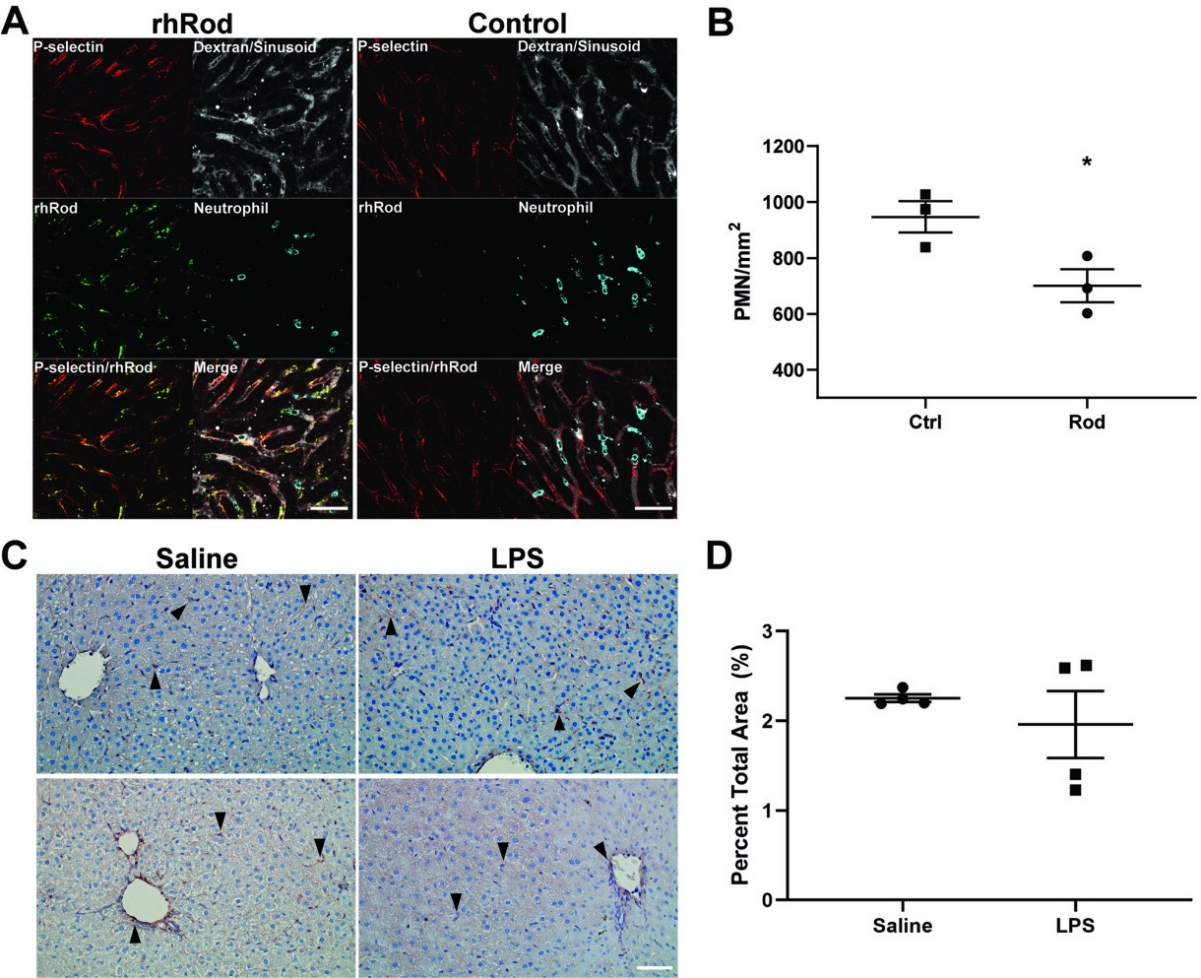
rhRod co-localizes with P-selectin and attenuates neutrophil accumulation in the liver sinusoids in endotoxemic mice. (A) Montage image from intravital microscopy of mouse liver sinusoids after intraperitoneal Atto 550-labeled rhRod (top) or control (bottom) followed by endotoxin injection. rhRod (green) and P-selectin (red) colocalize (yellow) along the sinusoid wall. Neutrophils were labeled with anti-Ly-6G antibody (cyan) and the sinusoids were identified using 150,000 Da-Dextran (gray). (B) rhRod administration decreased the number of firmly adherent (<1 cell body movement over 1 minute) neutrophils per vessel area in liver sinusoids. (C) Representative images of vimentin expression (brown; black arrows) with hematoxylin counterstain in the liver of mice 4 hours after saline or endotoxin intraperitoneal injection. (D) There was no difference in the percent area of vimentin staining between the two groups. Scale bar = 50 μm. *p<0.05.

P-selectin has been implicated in the pathogenesis of endotoxin-mediated liver injury in mice through the recruitment of leukocytes [10]. In our model, P-selectin was observed within the sinusoids independent of platelet accumulation (S3 Video). Because of the strong affinity of rhRod to P-sel/Fc, we tested whether Atto-550-labeled rhRod, given intraperitoneally 1 hour prior to endotoxin exposure, would colocalize with P-selectin in the liver sinusoids. Using intravital microscopy, we observed that rhRod bound to liver sinusoids and colocalized with P-selectin (Fig 6A). Endotoxemic mice receiving rhRod had a decreased number of firmly adherent neutrophils per vessel area than mice receiving dialysate control (700.6 ± 59.21 versus 947.1 ± 56 neutrophils/mm^2^, respectively, p<0.05; Fig 6B). When comparing the control and rhRod-treated groups, there were no differences in sinusoidal vessel density (155081 ± 13513 vs. 171029 ± 8214 μm^2^, respectively, p = 0.3703) or red blood cell flow velocity (148.5 ± 11.6 vs. 134.7 ± 21.3 μm/s, respectively; p = 0.599). These differences do not appear to be due to differences in liver vimentin expression 4 hours after endotoxin exposure (Fig 5C and 5D). These data corroborate our *in vitro* and *in silico* data in that rhRod binds to P-selectin *in vivo* to block neutrophil adhesion to inflamed endothelium.

## Discussion

Our previous study described that full-length recombinant vimentin binds to P-selectin to block leukocyte adhesion and decreases endotoxin-induced acute lung injury [14]. Our current data further refines those observations to localize the active binding regions on both recombinant vimentin and P-selectin. We are now demonstrating that this interaction between rhRod and P-selectin blocks leukocyte adhesion to inflamed endothelium. Our *in vitro* experiments comparing rhRod to rhVim confirms our previously published observations that rhVim blocks leukocyte adhesion through binding P-selectin with high affinity. Based on our previous report, one could suggest that vimentin would bind to PSGL-1 due to its binding to *N*-acetylglucosamine, a moiety present in PSGL-1 [32]. However, our bio-layer interferometry data testing rhVim and rhRod establish that they both bind to P-selectin, and not PSGL-1 in a calcium independent manner. The *in silico* and SPOT peptide array data provide more mechanistic insight into the regions to which rhRod interacts with P-selectin and *vice versa*. Indeed, our blocking assay using biolayer interferometry shows that rhRod blocks PSGL-1-P-selectin interactions at an IC50 of less than half of the K_D_ of PSGL-1 binding to P-selectin. Finally, our *in vivo* data using intravital microscopy in mouse livers during endotoxemia show rhRod colocalizing with P-selectin along the sinusoid walls and reducing the number of firmly adhered neutrophils as compared to control animals. These data support the notion that isolated rhRod is effective in blocking leukocyte adhesion through binding to P-selectin.

The combination of the SPOT peptide array data and *in silico* modelling indicate that rhRod apparently binds to P-selectin regions adjacent to the putative binding site for PSGL-1 [28]. Although the models displayed in Fig 3C represent the clusters with the lowest HADDOCK score (a composite of various binding energies and buried surface area) that are considered the most reliable, there were multiple possible configurations produced during simulation. Seven of the 10 lowest scoring clusters for 3TRT and 8 out of 10 for 1GK7 all showed the rod domain binding near the PSGL-1 binding site of P-selectin, giving confidence to the model. The remaining clusters all bound to an alternative site on P-selectin near the PSGL-1 binding site. These observations suggest that the inhibitory capacity of rhRod (and rhVim) is by directly blocking the PSGL-1 binding sites on P-selectin or by steric hindrance, or both. The observation of multiple possible rhRod-P-selectin configurations may explain the incomplete blockade of leukocyte adhesion to P-selectin, as some predicted configurations neither bind to the PSGL-1 binding site on P-selectin nor have rhRod positioned to sterically hinder a docking site.

One potential limitation of our *in silico* data is that the SPOT peptide array utilized 20-residue peptides per spot that may have different tertiary structures compared to full, intact proteins. Nonetheless, our BLI data measuring the K_D_ of rhVim and rhRod to P-selectin corroborates the SPOT array data. The SPOT array data identified the rod domain as the primary binding region of full-length vimentin to P-selectin, and the BLI data shows that rhRod, indeed, binds to P-selectin comparable to rhVim (Table 1). Furthermore, our observation that rhRod blocks PSGL-1-P-selectin interactions corroborate our *in silico* data in that rhRod is likely binding to the PSGL-1 binding site on P-selectin to inhibit leukocyte adhesion. Another limitation is the lack of the crystal structure for rhRod in complex with P-selectin, limiting us to employ known protein database (PDB) structures for both P-selectin (the EGF/lectin domain) and rhRod to inform our modelling. Importantly, the PDB structure for the rod domain of vimentin is incomplete since the entire rod domain has not been solved. Thus, our *in silico* data are assumptions based on our empirical SPOT peptide array data. Despite these limitations, the docking analysis identified a potential mechanism by which rhRod interacts with P-selectin to block leukocyte adhesion. Furthermore, only one alpha-helix was used for each docking run, while full length vimentin has a coiled-coil motif. Our model cannot predict whether vimentin can unfold to expose individual alpha-helices to P-selectin; however, when looking at the direct interactions between 3TRT and P-selectin in our model (Fig 3C, inset), the interactions occur between residues that are solvent exposed in 3TRT, rather than buried within the coiled-coil of vimentin, suggesting that unraveling is not a requirement for binding. The *in silico* prediction is also unable to determine whether large scale conformation changes take place upon binding to vimentin and P-selectin, however given the relative lack of structural alterations (RMSD 0.6 Å between Cα atoms between 1G1S and 1G1Q) on PSGL-1 binding to P-selectin (Fig 3A–3B), this would appear to be an unlikely scenario with vimentin and P-selectin. Limitations aside, using SPOT synthesis to map epitopes in combination with an information driven docking analysis appears novel in the literature, and can provide useful information in determining protein-protein interactions. The primary bottleneck in this technique is the reliance on deposited structures, however this technique is still more accessible than X-ray crystallography, NMR, and Cryo-EM for determining the structure of protein-protein interactions.

In our *in vitro* flow adhesion experiments, rhRod did not fully block leukocyte or isolated neutrophil adhesion to fibrin(ogen)-captured platelets, inflamed HUVEC, or P-selectin coated channels. These outcomes mimic our observations with full-length vimentin, in which we found that the addition of anti-ICAM-1 or -VCAM-1 to full-length vimentin further reduced, but did not fully abolish, neutrophil adhesion to IL-1β/IL-4 co-stimulated HUVEC [14]. This most likely represents the contribution of other cell adhesion molecule interactions (e.g. Mac-1-ICAM-1; Mac-1-GPIbα) in addition to P-selectin-PSGL-1 in our models [3, 33].

We detected P-selectin expression within 4 hours of endotoxin exposure in our intravital microscopy experiments. Furthermore, the majority of P-selectin was located within the sinusoids and much fewer in larger veins. This contrasts with a previously published report by Essani *et al*. that P-selectin was not found in the sinusoidal lining cells during endotoxemia in mice [34]. This discrepancy may due to the different antibodies utilized to detect P-selectin. We used a mouse anti-P-selectin monoclonal antibody (Psel.KO2.3) whereas that study used a rabbit polyclonal antibody. An earlier study by Doré *et al*., however, did observe the presence of P-selectin (called GMP-140) using a murine antibody (clone MD3) in the sinusoids of dog livers, although the authors contended that it could be due to staining either endothelial cells or platelets [35]. In our experiments, we observed that rhRod colocalizes with P-selectin in the sinusoids. Furthermore, we saw that P-selectin localization in the sinusoids was independent of platelets (S3 Video). These observations suggest that P-selectin is present in the hepatic sinusoids of mice and rhRod may block neutrophil adhesion *in vivo* through blocking P-selectin-PSGL-1 interactions.

Based on our data, recombinant rod domain of vimentin contains a major binding site for P-selectin. Moreover, recombinant rod domain is effective in blocking leukocyte adhesion to fibrin(ogen)-captured platelets and inflamed endothelium and in decreasing neutrophil recruitment into the liver sinusoids of endotoxemic mice. Therefore, rhRod may be a useful agent to attenuate inflammation in a variety of diseases, including sepsis.

## Supporting information

S1 Table SPOT peptide array layout of human vimentin (NP_003371). (DOCX)

S2 Table SPOT peptide array layout of human P-selectin (P16109.3). (DOCX)

S1 Fig Time-lapsed montage images from intravital microscopy of mouse livers in endotoxemic mice receiving (A) control or (B) Atto 550 labeled rhRod. The benefit of IVM is that it allows for differentiation between firmly adherent and mobile cells. The yellow arrows (B) point to the same PMN (cyan) that is initially immobile, but then quickly flows through the sinusoid (gray) after approximately 1 minute. rhRod (green) tends to colocalize (yellow) with P-selectin (red) along the sinusoids. The 1-minute video can be found in S6 Video (correlating to the montage). Scale bar = 50 μm.
(TIF)

S2 Fig Time-lapsed montage image of the FITC/dextran channel depicting RBC tracks from intravital microscopy of mouse liver. To estimate blood flow velocity in the sinusoids, RBC velocity was used. RBC were identified by negative contrast (black) within the dextran channel (white). The center of RBC were tracked frame by frame (colored lines) and used to calculate the RBC velocity within a segment. Scale bar = 50 μm.
(TIF)

S3 Fig Liver histology processing and quantification example. DAB-stained images were processed in FIJI/ImageJ. Each original image with hematoxylin counterstain (A) underwent colour deconvolution using the H DAB vector to isolate the DAB-only channel (B). To reduce background noise, all images underwent a Gaussian filter (radius 4; C). Finally, to determine the area stained by DAB (vimentin positive areas), a triangle auto-threshold was used (D).
(TIF)

S1 Methods Modelling of rhRod-P-selectin interactions. (DOCX)

S1 Video Time-compressed in vitro flow adhesion assay of mepacrine-labeled WBC flowing over IL-1β/IL-4 co-stimulated HUVEC. The image has been pseudocolored to aid visualization of leukocytes as compared to endothelial cells. Note the higher number of WBC in the (A) control versus the (B) rhRod arm. Scale bar = 50 μm.
(ZIP)

S2 Video 1-minute video correlating to the time-lapsed montage images in S1 Fig. Note the increased number of firmly adherent PMN (cyan) in the (A) control arm as compared to the (B) rhRod arm. Scale bar = 50 μm.
(ZIP)

S3 Video Intravital microscopy video in a baseline mouse of the central vein and liver sinusoids. Ten-second clips of the (A) central vein and (B) liver sinusoids. P-selectin (magenta) is primarily expressed in the sinusoids and is distinct from platelet (green) accumulation. Neutrophils are stained in cyan and vasculature in gray. Scale bar = 20 μm.
(ZIP)

## References

[1] Lam FW, Burns AR, Smith CW, Rumbaut RE. Platelets enhance neutrophil transendothelial migration via P-selectin glycoprotein ligand-1. Am J Physiol Heart Circ Physiol. 2011;300(2):H468–75. 10.1152/ajpheart.00491.2010 21169400 PMC3044064

[2] Jones DA, Abbassi O, McIntire LV, McEver RP, Smith CW. P-selectin mediates neutrophil rolling on histamine-stimulated endothelial cells. Biophys J. 1993;65(4):1560–9. 10.1016/S0006-3495(93)81195-0 7506064 PMC1225882

[3] Diacovo TG, Roth SJ, Buccola JM, Bainton DF, Springer TA. Neutrophil rolling, arrest, and transmigration across activated, surface-adherent platelets via sequential action of P-selectin and the beta 2-integrin CD11b/CD18. Blood. 1996;88(1):146–57. 8704169

[4] Konstantopoulos K, Neelamegham S, Burns AR, Hentzen E, Kansas GS, Snapp KR, et al Venous levels of shear support neutrophil-platelet adhesion and neutrophil aggregation in blood via P-selectin and beta2-integrin. Circulation. 1998;98(9):873–82. 10.1161/01.cir.98.9.873 9738642

[5] Moore KL, Stults NL, Diaz S, Smith DF, Cummings RD, Varki A, et al Identification of a specific glycoprotein ligand for P-selectin (CD62) on myeloid cells. J Cell Biol. 1992;118(2):445–56. 10.1083/jcb.118.2.445 1378449 PMC2290037

[6] Smith CW, Rothlein R, Hughes BJ, Mariscalco MM, Rudloff HE, Schmalstieg FC, et al Recognition of an endothelial determinant for CD 18-dependent human neutrophil adherence and transendothelial migration. J Clin Invest. 1988;82(5):1746–56. 10.1172/JCI113788 2903180 PMC442745

[7] Zarbock A, Singbartl K, Ley K. Complete reversal of acid-induced acute lung injury by blocking of platelet-neutrophil aggregation. J Clin Invest. 2006;116(12):3211–9. 10.1172/JCI29499 17143330 PMC1679711

[8] Asaduzzaman M, Lavasani S, Rahman M, Zhang S, Braun OO, Jeppsson B, et al Platelets support pulmonary recruitment of neutrophils in abdominal sepsis. Crit Care Med. 2009;37(4):1389–96. 10.1097/CCM.0b013e31819ceb71 19242347

[9] Kobashi H, Toshimori J, Yamamoto K. Sepsis-associated liver injury: Incidence, classification and the clinical significance. Hepatol Res. 2013;43(3):255–66. 10.1111/j.1872-034X.2012.01069.x 22971102

[10] Klintman D, Li X, Thorlacius H. Important role of P-selectin for leukocyte recruitment, hepatocellular injury, and apoptosis in endotoxemic mice. Clin Diagn Lab Immunol. 2004;11(1):56–62. 10.1128/cdli.11.1.56-62.2004 14715545 PMC321325

[11] Chernyatina AA, Nicolet S, Aebi U, Herrmann H, Strelkov SV. Atomic structure of the vimentin central alpha-helical domain and its implications for intermediate filament assembly. Proceedings of the National Academy of Sciences of the United States of America. 2012;109(34):13620–5. 10.1073/pnas.1206836109 22869704 PMC3427084

[12] Da Q, Behymer M, Correa JI, Vijayan KV, Cruz MA. Platelet adhesion involves a novel interaction between vimentin and von Willebrand factor under high shear stress. Blood. 2014;123(17):2715–21. 10.1182/blood-2013-10-530428 24642750 PMC3999756

[13] Mor-Vaknin N, Legendre M, Yu Y, Serezani CH, Garg SK, Jatzek A, et al Murine colitis is mediated by vimentin. Sci Rep. 2013;3:1045 10.1038/srep01045 23304436 PMC3540396

[14] Lam FW, Da Q, Guillory B, Cruz MA. Recombinant Human Vimentin Binds to P-Selectin and Blocks Neutrophil Capture and Rolling on Platelets and Endothelium. J Immunol. 2018;200(5):1718–26. 10.4049/jimmunol.1700784 29335256 PMC5821592

[15] Frank R. The SPOT-synthesis technique. Synthetic peptide arrays on membrane supports—principles and applications. J Immunol Methods. 2002;267(1):13–26. 10.1016/s0022-1759(02)00137-0 12135797

[16] van Zundert GCP, Rodrigues J, Trellet M, Schmitz C, Kastritis PL, Karaca E, et al The HADDOCK2.2 Web Server: User-Friendly Integrative Modeling of Biomolecular Complexes. J Mol Biol. 2016;428(4):720–5. 10.1016/j.jmb.2015.09.014 26410586

[17] Fernandes LS, Conde ID, Wayne Smith C, Kansas GS, Snapp KR, Bennet N, et al Platelet-monocyte complex formation: effect of blocking PSGL-1 alone, and in combination with alphaIIbbeta3 and alphaMbeta2, in coronary stenting. Thromb Res. 2003;111(3):171–7. 10.1016/j.thromres.2003.08.017 14678816

[18] Yao L, Pan J, Setiadi H, Patel KD, McEver RP. Interleukin 4 or oncostatin M induces a prolonged increase in P-selectin mRNA and protein in human endothelial cells. J Exp Med. 1996;184(1):81–92. 10.1084/jem.184.1.81 8691152 PMC2192668

[19] Da Q, Teruya M, Guchhait P, Teruya J, Olson JS, Cruz MA. Free hemoglobin increases von Willebrand factor-mediated platelet adhesion in vitro: implications for circulatory devices. Blood. 2015;126(20):2338–41. 10.1182/blood-2015-05-648030 26307534 PMC4643006

[20] Rueden CT, Schindelin J, Hiner MC, DeZonia BE, Walter AE, Arena ET, et al ImageJ2: ImageJ for the next generation of scientific image data. BMC Bioinformatics. 2017;18(1):529 10.1186/s12859-017-1934-z 29187165 PMC5708080

[21] Da Q, Derry PJ, Lam FW, Rumbaut RE. Fluorescent labeling of endogenous platelets for intravital microscopy: Effects on platelet function. Microcirculation. 2018;25(6):e12457 10.1111/micc.12457 29701894 PMC6204114

[22] Marques PE, Antunes MM, David BA, Pereira RV, Teixeira MM, Menezes GB. Imaging liver biology in vivo using conventional confocal microscopy. Nat Protoc. 2015;10(2):258–68. 10.1038/nprot.2015.006 25569332

[23] Park I, Choe K, Seo H, Hwang Y, Song E, Ahn J, et al Intravital imaging of a pulmonary endothelial surface layer in a murine sepsis model. Biomed Opt Express. 2018;9(5):2383–93. 10.1364/BOE.9.002383 29760995 PMC5946796

[24] Smal I, Meijering E, Dzyubachyk O. Methods for cell and particle tracking. Meth Enzymol. 2012;504:183–200. 10.1016/B978-0-12-391857-4.00009-4 22264535

[25] Law AMK, Yin JXM, Castillo L, Young AIJ, Piggin C, Rogers S, et al Andy's Algorithms: new automated digital image analysis pipelines for FIJI. Sci Rep. 2017;7(1):15717 10.1038/s41598-017-15885-6 29146920 PMC5691210

[26] Ruifrok AC, Johnston DA. Quantification of histochemical staining by color deconvolution. Anal Quant Cytol Histol. 2001;23(4):291–9. 11531144

[27] Zack GW, Rogers WE, Latt SA. Automatic measurement of sister chromatid exchange frequency. J Histochem Cytochem. 1977;25(7):741–53. 10.1177/25.7.70454 70454

[28] Somers WS, Tang J, Shaw GD, Camphausen RT. Insights into the molecular basis of leukocyte tethering and rolling revealed by structures of P- and E-selectin bound to SLe(X) and PSGL-1. Cell. 2000;103(3):467–79. 10.1016/s0092-8674(00)00138-0 11081633

[29] Pang AH, Obiero JM, Kulczyk AW, Sviripa VM, Tsodikov OV. A crystal structure of coil 1B of vimentin in the filamentous form provides a model of a high-order assembly of a vimentin filament. FEBS J. 2018;285(15):2888–99. 10.1111/febs.14585 29905014 PMC8022333

[30] Strelkov SV, Herrmann H, Geisler N, Wedig T, Zimbelmann R, Aebi U, et al Conserved segments 1A and 2B of the intermediate filament dimer: their atomic structures and role in filament assembly. Embo J. 2002;21(6):1255–66. 10.1093/emboj/21.6.1255 11889032 PMC125921

[31] Chernyatina AA, Strelkov SV. Stabilization of vimentin coil2 fragment via an engineered disulfide. J Struct Biol. 2012;177(1):46–53. 10.1016/j.jsb.2011.11.014 22119849

[32] Ise H, Kobayashi S, Goto M, Sato T, Kawakubo M, Takahashi M, et al Vimentin and desmin possess GlcNAc-binding lectin-like properties on cell surfaces. Glycobiology. 2010;20(7):843–64. 10.1093/glycob/cwq039 20332081

[33] Wang Y, Sakuma M, Chen Z, Ustinov V, Shi C, Croce K, et al Leukocyte engagement of platelet glycoprotein Ibalpha via the integrin Mac-1 is critical for the biological response to vascular injury. Circulation. 2005;112(19):2993–3000. 10.1161/CIRCULATIONAHA.105.571315 16260637

[34] Essani NA, Fisher MA, Simmons CA, Hoover JL, Farhood A, Jaeschke H. Increased P-selectin gene expression in the liver vasculature and its role in the pathophysiology of neutrophil-induced liver injury in murine endotoxin shock. J Leukoc Biol. 1998;63(3):288–96. 10.1002/jlb.63.3.288 9500515

[35] Dore M, Hawkins HK, Entman ML, Smith CW. Production of a monoclonal antibody against canine GMP-140 (P-selectin) and studies of its vascular distribution in canine tissues. Vet Pathol. 1993;30(3):213–22. 10.1177/030098589303000301 7687399

