# Supplementary material for: The vimentin rod domain blocks P-selectin-P-selectin glycoprotein ligand 1 interactions to attenuate leukocyte adhesion to inflamed endothelium
Source: PLoS One. 2020 Oct 13;15(10):e0240164. doi: 10.1371/journal.pone.0240164 (PMC7553327; doi:10.1371/journal.pone.0240164)
Supplement: S1 Table — (DOCX) [file pone.0240164.s001.docx]

| SPOT Peptide Array Layout: Human Vimentin (NP_003371) | | | |
| --- | --- | --- | --- |
| Spot No. | Location | Pred. MW (Da) | Amino Acid Sequence |
| 1 | A 1 | 2151.7 | MSTRSVSSSSYRRMFGGPGT |
| 2 | A 2 | 2091.6 | TRSVSSSSYRRMFGGPGTAS |
| 3 | A 3 | 2087.6 | SVSSSSYRRMFGGPGTASRP |
| 4 | A 4 | 2075.6 | SSSSYRRMFGGPGTASRPSS |
| 5 | A 5 | 2144.7 | SSYRRMFGGPGTASRPSSSR |
| 6 | A 6 | 2220.8 | YRRMFGGPGTASRPSSSRSY |
| 7 | A 7 | 2101.6 | RMFGGPGTASRPSSSRSYVT |
| 8 | A 8 | 2002.4 | FGGPGTASRPSSSRSYVTTS |
| 9 | A 9 | 2055.4 | GPGTASRPSSSRSYVTTSTR |
| 10 | A10 | 2165.5 | GTASRPSSSRSYVTTSTRTY |
| 11 | A11 | 2207.6 | ASRPSSSRSYVTTSTRTYSL |
| 12 | A12 | 2193.6 | RPSSSRSYVTTSTRTYSLGS |
| 13 | A13 | 2124.6 | SSSRSYVTTSTRTYSLGSAL |
| 14 | A14 | 2203.7 | SRSYVTTSTRTYSLGSALRP |
| 15 | A15 | 2148.6 | SYVTTSTRTYSLGSALRPST |
| 16 | A16 | 2141.6 | VTTSTRTYSLGSALRPSTSR |
| 17 | A17 | 2141.7 | TSTRTYSLGSALRPSTSRSL |
| 18 | A18 | 2187.8 | TRTYSLGSALRPSTSRSLYA |
| 19 | A19 | 2104.7 | TYSLGSALRPSTSRSLYASS |
| 20 | A20 | 1994.6 | SLGSALRPSTSRSLYASSPG |
| 21 | A21 | 1950.5 | GSALRPSTSRSLYASSPGGV |
| 22 | A22 | 2040.6 | ALRPSTSRSLYASSPGGVYA |
| 23 | A23 | 2113.6 | RPSTSRSLYASSPGGVYATR |
| 24 | A24 | 2034.5 | STSRSLYASSPGGVYATRSS |
| 25 | A25 | 2016.5 | SRSLYASSPGGVYATRSSAV |
| 26 | A26 | 2042.6 | SLYASSPGGVYATRSSAVRL |
| 27 | A27 | 2085.6 | YASSPGGVYATRSSAVRLRS |
| 28 | A28 | 2037.5 | SSPGGVYATRSSAVRLRSSV |
| 29 | A29 | 2017.5 | PGGVYATRSSAVRLRSSVPG |
| 30 | A30 | 2118.6 | GVYATRSSAVRLRSSVPGVR |
| 31 | B 1 | 2188.8 | YATRSSAVRLRSSVPGVRLL |
| 32 | B 2 | 2197.7 | TRSSAVRLRSSVPGVRLLQD |
| 33 | B 3 | 2126.6 | SSAVRLRSSVPGVRLLQDSV |
| 34 | B 4 | 2214.7 | AVRLRSSVPGVRLLQDSVDF |
| 35 | B 5 | 2244.8 | RLRSSVPGVRLLQDSVDFSL |
| 36 | B 6 | 2161.6 | RSSVPGVRLLQDSVDFSLAD |
| 37 | B 7 | 2102.6 | SVPGVRLLQDSVDFSLADAI |
| 38 | B 8 | 2131.6 | PGVRLLQDSVDFSLADAINT |
| 39 | B 9 | 2253.7 | VRLLQDSVDFSLADAINTEF |
| 40 | B10 | 2240.7 | LLQDSVDFSLADAINTEFKN |
| 41 | B11 | 2271.6 | QDSVDFSLADAINTEFKNTR |
| 42 | B12 | 2243.6 | SVDFSLADAINTEFKNTRTN |
| 43 | B13 | 2314.7 | DFSLADAINTEFKNTRTNEK |
| 44 | B14 | 2280.6 | SLADAINTEFKNTRTNEKVE |
| 45 | B15 | 2321.6 | ADAINTEFKNTRTNEKVELQ |
| 46 | B16 | 2377.7 | AINTEFKNTRTNEKVELQEL |
| 47 | B17 | 2422.6 | NTEFKNTRTNEKVELQELND |
| 48 | B18 | 2510.8 | EFKNTRTNEKVELQELNDRF |
| 49 | B19 | 2452.8 | FKNTRTNEKVELQELNDRFA |
| 50 | B20 | 2454.7 | NTRTNEKVELQELNDRFANY |
| 51 | B21 | 2467.8 | RTNEKVELQELNDRFANYID |
| 52 | B22 | 2437.8 | NEKVELQELNDRFANYIDKV |
| 53 | B23 | 2498 | KVELQELNDRFANYIDKVRF |
| 54 | B24 | 2513 | ELQELNDRFANYIDKVRFLE |
| 55 | B25 | 2526.9 | QELNDRFANYIDKVRFLEQQ |
| 56 | B26 | 2512 | LNDRFANYIDKVRFLEQQNK |
| 57 | B27 | 2511.1 | DRFANYIDKVRFLEQQNKIL |
| 58 | B28 | 2424.1 | FANYIDKVRFLEQQNKILLA |
| 59 | B29 | 2448.1 | NYIDKVRFLEQQNKILLAEL |
| 60 | B30 | 2428 | IDKVRFLEQQNKILLAELEQ |
| 61 | C 1 | 2441.1 | KVRFLEQQNKILLAELEQLK |
| 62 | C 2 | 2399 | RFLEQQNKILLAELEQLKGQ |
| 63 | C 3 | 2280.9 | LEQQNKILLAELEQLKGQGK |
| 64 | C 4 | 2281.9 | QQNKILLAELEQLKGQGKSR |
| 65 | C 5 | 2196 | NKILLAELEQLKGQGKSRLG |
| 66 | C 6 | 2182 | ILLAELEQLKGQGKSRLGDL |
| 67 | C 7 | 2247.9 | LAELEQLKGQGKSRLGDLYE |
| 68 | C 8 | 2321.8 | ELEQLKGQGKSRLGDLYEEE |
| 69 | C 9 | 2366.9 | EQLKGQGKSRLGDLYEEEMR |
| 70 | C10 | 2352 | LKGQGKSRLGDLYEEEMREL |
| 71 | C11 | 2423 | GQGKSRLGDLYEEEMRELRR |
| 72 | C12 | 2465 | GKSRLGDLYEEEMRELRRQV |
| 73 | C13 | 2522.9 | SRLGDLYEEEMRELRRQVDQ |
| 74 | C14 | 2493.9 | LGDLYEEEMRELRRQVDQLT |
| 75 | C15 | 2552.8 | DLYEEEMRELRRQVDQLTND |
| 76 | C16 | 2523.8 | YEEEMRELRRQVDQLTNDKA |
| 77 | C17 | 2486.8 | EEMRELRRQVDQLTNDKARV |
| 78 | C18 | 2456.8 | MRELRRQVDQLTNDKARVEV |
| 79 | C19 | 2454.7 | ELRRQVDQLTNDKARVEVER |
| 80 | C20 | 2441.6 | RRQVDQLTNDKARVEVERDN |
| 81 | C21 | 2313.5 | QVDQLTNDKARVEVERDNLA |
| 82 | C22 | 2330.5 | DQLTNDKARVEVERDNLAED |
| 83 | C23 | 2331.7 | LTNDKARVEVERDNLAEDIM |
| 84 | C24 | 2386.8 | NDKARVEVERDNLAEDIMRL |
| 85 | C25 | 2442.9 | KARVEVERDNLAEDIMRLRE |
| 86 | C26 | 2485 | RVEVERDNLAEDIMRLREKL |
| 87 | C27 | 2486.9 | EVERDNLAEDIMRLREKLQE |
| 88 | C28 | 2519 | ERDNLAEDIMRLREKLQEEM |
| 89 | C29 | 2475 | DNLAEDIMRLREKLQEEMLQ |
| 90 | C30 | 2531.1 | LAEDIMRLREKLQEEMLQRE |
| 91 | D 1 | 2547 | EDIMRLREKLQEEMLQREEA |
| 92 | D 2 | 2546 | IMRLREKLQEEMLQREEAEN |
| 93 | D 3 | 2515.9 | RLREKLQEEMLQREEAENTL |
| 94 | D 4 | 2461.7 | REKLQEEMLQREEAENTLQS |
| 95 | D 5 | 2479.8 | KLQEEMLQREEAENTLQSFR |
| 96 | D 6 | 2481.6 | QEEMLQREEAENTLQSFRQD |
| 97 | D 7 | 2438.6 | EMLQREEAENTLQSFRQDVD |
| 98 | D 8 | 2363.5 | LQREEAENTLQSFRQDVDNA |
| 99 | D 9 | 2322.5 | REEAENTLQSFRQDVDNASL |
| 100 | D10 | 2264.5 | EAENTLQSFRQDVDNASLAR |
| 101 | D11 | 2292.6 | ENTLQSFRQDVDNASLARLD |
| 102 | D12 | 2291.7 | TLQSFRQDVDNASLARLDLE |
| 103 | D13 | 2361.8 | QSFRQDVDNASLARLDLERK |
| 104 | D14 | 2374.8 | FRQDVDNASLARLDLERKVE |
| 105 | D15 | 2271.7 | QDVDNASLARLDLERKVESL |
| 106 | D16 | 2285.7 | VDNASLARLDLERKVESLQE |
| 107 | D17 | 2313.8 | NASLARLDLERKVESLQEEI |
| 108 | D18 | 2346.9 | SLARLDLERKVESLQEEIAF |
| 109 | D19 | 2388 | ARLDLERKVESLQEEIAFLK |
| 110 | D20 | 2402.1 | LDLERKVESLQEEIAFLKKL |
| 111 | D21 | 2440 | LERKVESLQEEIAFLKKLHE |
| 112 | D22 | 2455.9 | RKVESLQEEIAFLKKLHEEE |
| 113 | D23 | 2412.8 | VESLQEEIAFLKKLHEEEIQ |
| 114 | D24 | 2426.9 | SLQEEIAFLKKLHEEEIQEL |
| 115 | D25 | 2425.8 | QEEIAFLKKLHEEEIQELQA |
| 116 | D26 | 2409.9 | EIAFLKKLHEEEIQELQAQI |
| 117 | D27 | 2424.8 | AFLKKLHEEEIQELQAQIQE |
| 118 | D28 | 2471.7 | LKKLHEEEIQELQAQIQEQH |
| 119 | D29 | 2457.5 | KLHEEEIQELQAQIQEQHVQ |
| 120 | D30 | 2444.4 | HEEEIQELQAQIQEQHVQID |
| 121 | E 1 | 2392.4 | EEIQELQAQIQEQHVQIDVD |
| 122 | E 2 | 2320.4 | IQELQAQIQEQHVQIDVDVS |
| 123 | E 3 | 2304.4 | ELQAQIQEQHVQIDVDVSKP |
| 124 | E 4 | 2290.4 | QAQIQEQHVQIDVDVSKPDL |
| 125 | E 5 | 2263.4 | QIQEQHVQIDVDVSKPDLTA |
| 126 | E 6 | 2206.4 | QEQHVQIDVDVSKPDLTAAL |
| 127 | E 7 | 2220.5 | QHVQIDVDVSKPDLTAALRD |
| 128 | E 8 | 2210.6 | VQIDVDVSKPDLTAALRDVR |
| 129 | E 9 | 2239.6 | IDVDVSKPDLTAALRDVRQQ |
| 130 | E10 | 2303.6 | VDVSKPDLTAALRDVRQQYE |
| 131 | E11 | 2275.6 | VSKPDLTAALRDVRQQYESV |
| 132 | E12 | 2231.6 | KPDLTAALRDVRQQYESVAA |
| 133 | E13 | 2248.6 | DLTAALRDVRQQYESVAAKN |
| 134 | E14 | 2261.6 | TAALRDVRQQYESVAAKNLQ |
| 135 | E15 | 2289.6 | ALRDVRQQYESVAAKNLQEA |
| 136 | E16 | 2363.5 | RDVRQQYESVAAKNLQEAEE |
| 137 | E17 | 2441.6 | VRQQYESVAAKNLQEAEEWY |
| 138 | E18 | 2401.6 | QQYESVAAKNLQEAEEWYKS |
| 139 | E19 | 2420.8 | YESVAAKNLQEAEEWYKSKF |
| 140 | E20 | 2314.7 | SVAAKNLQEAEEWYKSKFAD |
| 141 | E21 | 2328.8 | AAKNLQEAEEWYKSKFADLS |
| 142 | E22 | 2386.8 | KNLQEAEEWYKSKFADLSEA |
| 143 | E23 | 2329.7 | LQEAEEWYKSKFADLSEAAN |
| 144 | E24 | 2358.7 | EAEEWYKSKFADLSEAANRN |
| 145 | E25 | 2387.7 | EEWYKSKFADLSEAANRNND |
| 146 | E26 | 2313.8 | WYKSKFADLSEAANRNNDAL |
| 147 | E27 | 2248.7 | KSKFADLSEAANRNNDALRQ |
| 148 | E28 | 2232.7 | KFADLSEAANRNNDALRQAK |
| 149 | E29 | 2214.5 | ADLSEAANRNNDALRQAKQE |
| 150 | E30 | 2216.5 | LSEAANRNNDALRQAKQEST |
| 151 | F 1 | 2308.5 | EAANRNNDALRQAKQESTEY |
| 152 | F 2 | 2420.7 | ANRNNDALRQAKQESTEYRR |
| 153 | F 3 | 2462.7 | RNNDALRQAKQESTEYRRQV |
| 154 | F 4 | 2407.6 | NDALRQAKQESTEYRRQVQS |
| 155 | F 5 | 2392.7 | ALRQAKQESTEYRRQVQSLT |
| 156 | F 6 | 2440.6 | RQAKQESTEYRRQVQSLTCE |
| 157 | F 7 | 2370.5 | AKQESTEYRRQVQSLTCEVD |
| 158 | F 8 | 2355.5 | QESTEYRRQVQSLTCEVDAL |
| 159 | F 9 | 2283.6 | STEYRRQVQSLTCEVDALKG |
| 160 | F10 | 2310.6 | EYRRQVQSLTCEVDALKGTN |
| 161 | F11 | 2234.5 | RRQVQSLTCEVDALKGTNES |
| 162 | F12 | 2164.4 | QVQSLTCEVDALKGTNESLE |
| 163 | F13 | 2221.5 | QSLTCEVDALKGTNESLERQ |
| 164 | F14 | 2293.7 | LTCEVDALKGTNESLERQMR |
| 165 | F15 | 2339.7 | CEVDALKGTNESLERQMREM |
| 166 | F16 | 2365.7 | VDALKGTNESLERQMREMEE |
| 167 | F17 | 2412.8 | ALKGTNESLERQMREMEENF |
| 168 | F18 | 2398.7 | KGTNESLERQMREMEENFAV |
| 169 | F19 | 2413.6 | TNESLERQMREMEENFAVEA |
| 170 | F20 | 2383.6 | ESLERQMREMEENFAVEAAN |
| 171 | F21 | 2458.7 | LERQMREMEENFAVEAANYQ |
| 172 | F22 | 2432.6 | RQMREMEENFAVEAANYQDT |
| 173 | F23 | 2318.6 | MREMEENFAVEAANYQDTIG |
| 174 | F24 | 2300.6 | EMEENFAVEAANYQDTIGRL |
| 175 | F25 | 2283.5 | EENFAVEAANYQDTIGRLQD |
| 176 | F26 | 2267.6 | NFAVEAANYQDTIGRLQDEI |
| 177 | F27 | 2248.5 | AVEAANYQDTIGRLQDEIQN |
| 178 | F28 | 2337.7 | EAANYQDTIGRLQDEIQNMK |
| 179 | F29 | 2395.7 | ANYQDTIGRLQDEIQNMKEE |
| 180 | F30 | 2412.8 | YQDTIGRLQDEIQNMKEEMA |
| 181 | G 1 | 2414.8 | DTIGRLQDEIQNMKEEMARH |
| 182 | G 2 | 2468 | IGRLQDEIQNMKEEMARHLR |
| 183 | G 3 | 2590 | RLQDEIQNMKEEMARHLREY |
| 184 | G 4 | 2563.8 | QDEIQNMKEEMARHLREYQD |
| 185 | G 5 | 2547 | EIQNMKEEMARHLREYQDLL |
| 186 | G 6 | 2517.9 | QNMKEEMARHLREYQDLLNV |
| 187 | G 7 | 2535.1 | MKEEMARHLREYQDLLNVKM |
| 188 | G 8 | 2460 | EEMARHLREYQDLLNVKMAL |
| 189 | G 9 | 2430.1 | MARHLREYQDLLNVKMALDI |
| 190 | G10 | 2470.1 | RHLREYQDLLNVKMALDIEI |
| 191 | G11 | 2349 | LREYQDLLNVKMALDIEIAT |
| 192 | G12 | 2399 | EYQDLLNVKMALDIEIATYR |
| 193 | G13 | 2348.1 | QDLLNVKMALDIEIATYRKL |
| 194 | G14 | 2347.2 | LLNVKMALDIEIATYRKLLE |
| 195 | G15 | 2307 | NVKMALDIEIATYRKLLEGE |
| 196 | G16 | 2310 | KMALDIEIATYRKLLEGEES |
| 197 | G17 | 2320 | ALDIEIATYRKLLEGEESRI |
| 198 | G18 | 2336 | DIEIATYRKLLEGEESRISL |
| 199 | G19 | 2318 | EIATYRKLLEGEESRISLPL |
| 200 | G20 | 2286.9 | ATYRKLLEGEESRISLPLPN |
| 201 | G21 | 2349 | YRKLLEGEESRISLPLPNFS |
| 202 | G22 | 2229.9 | KLLEGEESRISLPLPNFSSL |
| 203 | G23 | 2215.8 | LEGEESRISLPLPNFSSLNL |
| 204 | G24 | 2258.8 | GEESRISLPLPNFSSLNLRE |
| 205 | G25 | 2287.8 | ESRISLPLPNFSSLNLRETN |
| 206 | G26 | 2299.9 | RISLPLPNFSSLNLRETNLD |
| 207 | G27 | 2230.8 | SLPLPNFSSLNLRETNLDSL |
| 208 | G28 | 2240.8 | PLPNFSSLNLRETNLDSLPL |
| 209 | G29 | 2244.7 | PNFSSLNLRETNLDSLPLVD |
| 210 | G30 | 2271.7 | FSSLNLRETNLDSLPLVDTH |
| 211 | H 1 | 2252.7 | SLNLRETNLDSLPLVDTHSK |
| 212 | H 2 | 2309.7 | NLRETNLDSLPLVDTHSKRT |
| 213 | H 3 | 2308.8 | RETNLDSLPLVDTHSKRTLL |
| 214 | H 4 | 2264.9 | TNLDSLPLVDTHSKRTLLIK |
| 215 | H 5 | 2249.9 | LDSLPLVDTHSKRTLLIKTV |
| 216 | H 6 | 2251.8 | SLPLVDTHSKRTLLIKTVET |
| 217 | H 7 | 2322.8 | PLVDTHSKRTLLIKTVETRD |
| 218 | H 8 | 2297.7 | VDTHSKRTLLIKTVETRDGQ |
| 219 | H 9 | 2295.8 | THSKRTLLIKTVETRDGQVI |
| 220 | H10 | 2300.8 | SKRTLLIKTVETRDGQVINE |
| 221 | H11 | 2273.7 | RTLLIKTVETRDGQVINETS |
| 222 | H12 | 2281.6 | LLIKTVETRDGQVINETSQH |
| 223 | H13 | 2307.4 | IKTVETRDGQVINETSQHHD |
| 224 | H14 | 2294.3 | TVETRDGQVINETSQHHDDL |

**S2 Table. Layout of the SPOT Peptide Array of Human Vimentin**
