# Supplementary material for: The vimentin rod domain blocks P-selectin-P-selectin glycoprotein ligand 1 interactions to attenuate leukocyte adhesion to inflamed endothelium
Source: PLoS One. 2020 Oct 13;15(10):e0240164. doi: 10.1371/journal.pone.0240164 (PMC7553327; doi:10.1371/journal.pone.0240164)
Supplement: S2 Table — (DOCX) [file pone.0240164.s002.docx]

| SPOT Peptide Array Layout: Human P-selectin (UniProt P16109.3) | | | |
| --- | --- | --- | --- |
| Spot No. | Location | Pred. MW (Da) | Amino Acid Sequence |
|  | A23 | 2371 | MANCQIAILYQRFQRVVFGI |
|  | A24 | 2383 | CQIAILYQRFQRVVFGISQL |
|  | A25 | 2402.1 | AILYQRFQRVVFGISQLLCF |
|  | A26 | 2376 | YQRFQRVVFGISQLLCFSAL |
|  | A27 | 2257.9 | FQRVVFGISQLLCFSALISE |
|  | A28 | 2154.8 | VVFGISQLLCFSALISELTN |
|  | A29 | 2194.8 | GISQLLCFSALISELTNQKE |
|  | A30 | 2178.7 | QLLCFSALISELTNQKEVAA |
|  | B 1 | 2274.7 | CFSALISELTNQKEVAAWTY |
|  | B 2 | 2324.7 | ALISELTNQKEVAAWTYHYS |
|  | B 3 | 2327.6 | SELTNQKEVAAWTYHYSTKA |
|  | B 4 | 2434.7 | TNQKEVAAWTYHYSTKAYSW |
|  | B 5 | 2405.8 | KEVAAWTYHYSTKAYSWNIS |
|  | B 6 | 2497 | AAWTYHYSTKAYSWNISRKY |
|  | B 7 | 2513.9 | TYHYSTKAYSWNISRKYCQN |
|  | B 8 | 2533 | YSTKAYSWNISRKYCQNRYT |
|  | B 9 | 2509 | KAYSWNISRKYCQNRYTDLV |
|  | B10 | 2458.9 | SWNISRKYCQNRYTDLVAIQ |
|  | B11 | 2427.9 | ISRKYCQNRYTDLVAIQNKN |
|  | B12 | 2428.8 | KYCQNRYTDLVAIQNKNEID |
|  | B13 | 2424.8 | QNRYTDLVAIQNKNEIDYLN |
|  | B14 | 2366.9 | YTDLVAIQNKNEIDYLNKVL |
|  | B15 | 2411 | LVAIQNKNEIDYLNKVLPYY |
|  | B16 | 2465 | IQNKNEIDYLNKVLPYYSSY |
|  | B17 | 2572.2 | KNEIDYLNKVLPYYSSYYWI |
|  | B18 | 2527.3 | IDYLNKVLPYYSSYYWIGIR |
|  | B19 | 2492.2 | LNKVLPYYSSYYWIGIRKNN |
|  | B20 | 2552.2 | VLPYYSSYYWIGIRKNNKTW |
|  | B21 | 2629.2 | YYSSYYWIGIRKNNKTWTWV |
|  | B22 | 2502.1 | SYYWIGIRKNNKTWTWVGTK |
|  | B23 | 2401.1 | WIGIRKNNKTWTWVGTKKAL |
|  | B24 | 2388.9 | IRKNNKTWTWVGTKKALTNE |
|  | B25 | 2305.6 | NNKTWTWVGTKKALTNEAEN |
|  | B26 | 2321.6 | TWTWVGTKKALTNEAENWAD |
|  | B27 | 2273.5 | WVGTKKALTNEAENWADNEP |
|  | B28 | 2287.5 | TKKALTNEAENWADNEPNNK |
|  | B29 | 2314.4 | ALTNEAENWADNEPNNKRNN |
|  | B30 | 2376.3 | NEAENWADNEPNNKRNNEDC |
|  | C 1 | 2403.4 | ENWADNEPNNKRNNEDCVEI |
|  | C 2 | 2378.6 | ADNEPNNKRNNEDCVEIYIK |
|  | C 3 | 2349.6 | EPNNKRNNEDCVEIYIKSPS |
|  | C 4 | 2234.6 | NKRNNEDCVEIYIKSPSAPG |
|  | C 5 | 2264.6 | NNEDCVEIYIKSPSAPGKWN |
|  | C 6 | 2288.6 | DCVEIYIKSPSAPGKWNDEH |
|  | C 7 | 2315.8 | EIYIKSPSAPGKWNDEHCLK |
|  | C 8 | 2303.8 | IKSPSAPGKWNDEHCLKKKH |
|  | C 9 | 2262.7 | PSAPGKWNDEHCLKKKHALC |
|  | C10 | 2342.8 | PGKWNDEHCLKKKHALCYTA |
|  | C11 | 2378.7 | WNDEHCLKKKHALCYTASCQ |
|  | C12 | 2296.7 | EHCLKKKHALCYTASCQDMS |
|  | C13 | 2245.8 | LKKKHALCYTASCQDMSCSK |
|  | C14 | 2190.5 | KHALCYTASCQDMSCSKQGE |
|  | C15 | 2199.5 | LCYTASCQDMSCSKQGECLE |
|  | C16 | 2091.4 | TASCQDMSCSKQGECLETIG |
|  | C17 | 2210.5 | CQDMSCSKQGECLETIGNYT |
|  | C18 | 2157.5 | MSCSKQGECLETIGNYTCSC |
|  | C19 | 2153.5 | SKQGECLETIGNYTCSCYPG |
|  | C20 | 2177.6 | GECLETIGNYTCSCYPGFYG |
|  | C21 | 2217.6 | LETIGNYTCSCYPGFYGPEC |
|  | C22 | 2265.6 | IGNYTCSCYPGFYGPECEYV |
|  | C23 | 2369.6 | YTCSCYPGFYGPECEYVREC |
|  | C24 | 2301.6 | SCYPGFYGPECEYVRECGEL |
|  | C25 | 2287.6 | PGFYGPECEYVRECGELELP |
|  | C26 | 2350.5 | YGPECEYVRECGELELPQHV |
|  | C27 | 2391.6 | ECEYVRECGELELPQHVLMN |
|  | C28 | 2357.6 | YVRECGELELPQHVLMNCSH |
|  | C29 | 2206.5 | ECGELELPQHVLMNCSHPLG |
|  | C30 | 2265.6 | ELELPQHVLMNCSHPLGNFS |
|  | D 1 | 2242.6 | LPQHVLMNCSHPLGNFSFNS |
|  | D 2 | 2222.5 | HVLMNCSHPLGNFSFNSQCS |
|  | D 3 | 2260.5 | MNCSHPLGNFSFNSQCSFHC |
|  | D 4 | 2185.4 | SHPLGNFSFNSQCSFHCTDG |
|  | D 5 | 2254.5 | LGNFSFNSQCSFHCTDGYQV |
|  | D 6 | 2238.4 | FSFNSQCSFHCTDGYQVNGP |
|  | D 7 | 2185.4 | NSQCSFHCTDGYQVNGPSKL |
|  | D 8 | 2201.5 | CSFHCTDGYQVNGPSKLECL |
|  | D 9 | 2079.4 | HCTDGYQVNGPSKLECLASG |
|  | D10 | 2138.6 | DGYQVNGPSKLECLASGIWT |
|  | D11 | 2142.6 | QVNGPSKLECLASGIWTNKP |
|  | D12 | 2129.6 | GPSKLECLASGIWTNKPPQC |
|  | D13 | 2143.7 | KLECLASGIWTNKPPQCLAA |
|  | D14 | 2101.5 | CLASGIWTNKPPQCLAAQCP |
|  | D15 | 2152.6 | SGIWTNKPPQCLAAQCPPLK |
|  | D16 | 2234.6 | WTNKPPQCLAAQCPPLKIPE |
|  | D17 | 2160.6 | KPPQCLAAQCPPLKIPERGN |
|  | D18 | 2173.6 | QCLAAQCPPLKIPERGNMTC |
|  | D19 | 2166.6 | AAQCPPLKIPERGNMTCLHS |
|  | D20 | 2166.7 | CPPLKIPERGNMTCLHSAKA |
|  | D21 | 2281.8 | LKIPERGNMTCLHSAKAFQH |
|  | D22 | 2229.5 | PERGNMTCLHSAKAFQHQSS |
|  | D23 | 2184.5 | GNMTCLHSAKAFQHQSSCSF |
|  | D24 | 2201.4 | TCLHSAKAFQHQSSCSFSCE |
|  | D25 | 2217.4 | HSAKAFQHQSSCSFSCEEGF |
|  | D26 | 2205.5 | KAFQHQSSCSFSCEEGFALV |
|  | D27 | 2142.3 | QHQSSCSFSCEEGFALVGPE |
|  | D28 | 2075.3 | SSCSFSCEEGFALVGPEVVQ |
|  | D29 | 2073.3 | SFSCEEGFALVGPEVVQCTA |
|  | D30 | 1995.2 | CEEGFALVGPEVVQCTASGV |
|  | E 1 | 1992.3 | GFALVGPEVVQCTASGVWTA |
|  | E 2 | 1982.2 | LVGPEVVQCTASGVWTAPAP |
|  | E 3 | 2043.2 | PEVVQCTASGVWTAPAPVCK |
|  | E 4 | 2016.2 | VQCTASGVWTAPAPVCKAVQ |
|  | E 5 | 2054.2 | TASGVWTAPAPVCKAVQCQH |
|  | E 6 | 2108.3 | GVWTAPAPVCKAVQCQHLEA |
|  | E 7 | 2079.2 | TAPAPVCKAVQCQHLEAPSE |
|  | E 8 | 2099.3 | APVCKAVQCQHLEAPSEGTM |
|  | E 9 | 2149.3 | CKAVQCQHLEAPSEGTMDCV |
|  | E10 | 2194.3 | VQCQHLEAPSEGTMDCVHPL |
|  | E11 | 2183.4 | QHLEAPSEGTMDCVHPLTAF |
|  | E12 | 2096.4 | EAPSEGTMDCVHPLTAFAYG |
|  | E13 | 2076.4 | SEGTMDCVHPLTAFAYGSSC |
|  | E14 | 2207.6 | TMDCVHPLTAFAYGSSCKFE |
|  | E15 | 2188.5 | CVHPLTAFAYGSSCKFECQP |
|  | E16 | 2225.7 | PLTAFAYGSSCKFECQPGYR |
|  | E17 | 2226.7 | AFAYGSSCKFECQPGYRVRG |
|  | E18 | 2296.8 | YGSSCKFECQPGYRVRGLDM |
|  | E19 | 2361.9 | SCKFECQPGYRVRGLDMLRC |
|  | E20 | 2358.9 | FECQPGYRVRGLDMLRCIDS |
|  | E21 | 2359.9 | QPGYRVRGLDMLRCIDSGHW |
|  | E22 | 2332.9 | YRVRGLDMLRCIDSGHWSAP |
|  | E23 | 2225.8 | RGLDMLRCIDSGHWSAPLPT |
|  | E24 | 2202.6 | DMLRCIDSGHWSAPLPTCEA |
|  | E25 | 2146.5 | RCIDSGHWSAPLPTCEAISC |
|  | E26 | 2113.4 | DSGHWSAPLPTCEAISCEPL |
|  | E27 | 2167.4 | HWSAPLPTCEAISCEPLESP |
|  | E28 | 2050.3 | APLPTCEAISCEPLESPVHG |
|  | E29 | 2102.3 | PTCEAISCEPLESPVHGSMD |
|  | E30 | 2088.3 | EAISCEPLESPVHGSMDCSP |
|  | F 1 | 2131.4 | SCEPLESPVHGSMDCSPSLR |
|  | F 2 | 2158.5 | PLESPVHGSMDCSPSLRAFQ |
|  | F 3 | 2198.5 | SPVHGSMDCSPSLRAFQYDT |
|  | F 4 | 2219.5 | HGSMDCSPSLRAFQYDTNCS |
|  | F 5 | 2344.7 | MDCSPSLRAFQYDTNCSFRC |
|  | F 6 | 2252.6 | SPSLRAFQYDTNCSFRCAEG |
|  | F 7 | 2372.9 | LRAFQYDTNCSFRCAEGFML |
|  | F 8 | 2316.8 | FQYDTNCSFRCAEGFMLRGA |
|  | F 9 | 2205.7 | DTNCSFRCAEGFMLRGADIV |
|  | F10 | 2249.8 | CSFRCAEGFMLRGADIVRCD |
|  | F11 | 2196.8 | RCAEGFMLRGADIVRCDNLG |
|  | F12 | 2281.8 | EGFMLRGADIVRCDNLGQWT |
|  | F13 | 2187.7 | MLRGADIVRCDNLGQWTAPA |
|  | F14 | 2086.4 | GADIVRCDNLGQWTAPAPVC |
|  | F15 | 2155.5 | IVRCDNLGQWTAPAPVCQAL |
|  | F16 | 2146.3 | CDNLGQWTAPAPVCQALQCQ |
|  | F17 | 2139.4 | LGQWTAPAPVCQALQCQDLP |
|  | F18 | 2151.3 | WTAPAPVCQALQCQDLPVPN |
|  | F19 | 2149.3 | PAPVCQALQCQDLPVPNEAR |
|  | F20 | 2200.3 | VCQALQCQDLPVPNEARVNC |
|  | F21 | 2191.3 | ALQCQDLPVPNEARVNCSHP |
|  | F22 | 2154.3 | CQDLPVPNEARVNCSHPFGA |
|  | F23 | 2274.6 | LPVPNEARVNCSHPFGAFRY |
|  | F24 | 2279.5 | PNEARVNCSHPFGAFRYQSV |
|  | F25 | 2276.6 | ARVNCSHPFGAFRYQSVCSF |
|  | F26 | 2268.5 | NCSHPFGAFRYQSVCSFTCN |
|  | F27 | 2263.6 | HPFGAFRYQSVCSFTCNEGL |
|  | F28 | 2207.7 | GAFRYQSVCSFTCNEGLLLV |
|  | F29 | 2147.6 | RYQSVCSFTCNEGLLLVGAS |
|  | F30 | 2040.5 | SVCSFTCNEGLLLVGASVLQ |
|  | G 1 | 2038.6 | SFTCNEGLLLVGASVLQCLA |
|  | G 2 | 1975.5 | CNEGLLLVGASVLQCLATGN |
|  | G 3 | 2016.6 | GLLLVGASVLQCLATGNWNS |
|  | G 4 | 2026.4 | LVGASVLQCLATGNWNSVPP |
|  | G 5 | 2117.3 | ASVLQCLATGNWNSVPPECQ |
|  | G 6 | 2141.4 | LQCLATGNWNSVPPECQAIP |
|  | G 7 | 2098.3 | LATGNWNSVPPECQAIPCTP |
|  | G 8 | 2126.4 | GNWNSVPPECQAIPCTPLLS |
|  | G 9 | 2108.3 | NSVPPECQAIPCTPLLSPQN |
|  | G10 | 2097.4 | PPECQAIPCTPLLSPQNGTM |
|  | G11 | 2077.4 | CQAIPCTPLLSPQNGTMTCV |
|  | G12 | 2113.5 | IPCTPLLSPQNGTMTCVQPL |
|  | G13 | 2031.4 | TPLLSPQNGTMTCVQPLGSS |
|  | G14 | 2098.5 | LSPQNGTMTCVQPLGSSSYK |
|  | G15 | 2092.4 | QNGTMTCVQPLGSSSYKSTC |
|  | G16 | 2181.6 | TMTCVQPLGSSSYKSTCQFI |
|  | G17 | 2195.5 | CVQPLGSSSYKSTCQFICDE |
|  | G18 | 2172.6 | PLGSSSYKSTCQFICDEGYS |
|  | G19 | 2162.6 | SSSYKSTCQFICDEGYSLSG |
|  | G20 | 2283.7 | YKSTCQFICDEGYSLSGPER |
|  | G21 | 2236.6 | TCQFICDEGYSLSGPERLDC |
|  | G22 | 2248.7 | FICDEGYSLSGPERLDCTRS |
|  | G23 | 2284.7 | DEGYSLSGPERLDCTRSGRW |
|  | G24 | 2286.7 | YSLSGPERLDCTRSGRWTDS |
|  | G25 | 2248.6 | SGPERLDCTRSGRWTDSPPM |
|  | G26 | 2310.6 | ERLDCTRSGRWTDSPPMCEA |
|  | G27 | 2256.6 | DCTRSGRWTDSPPMCEAIKC |
|  | G28 | 2276.7 | RSGRWTDSPPMCEAIKCPEL |
|  | G29 | 2291.7 | RWTDSPPMCEAIKCPELFAP |
|  | G30 | 2162.5 | DSPPMCEAIKCPELFAPEQG |
|  | H 1 | 2178.6 | PMCEAIKCPELFAPEQGSLD |
|  | H 2 | 2152.5 | EAIKCPELFAPEQGSLDCSD |
|  | H 3 | 2153.5 | KCPELFAPEQGSLDCSDTRG |
|  | H 4 | 2215.5 | ELFAPEQGSLDCSDTRGEFN |
|  | H 5 | 2069.3 | APEQGSLDCSDTRGEFNVGS |
|  | H 6 | 2113.3 | QGSLDCSDTRGEFNVGSTCH |
|  | H 7 | 2178.4 | LDCSDTRGEFNVGSTCHFSC |
|  | H 8 | 2133.3 | SDTRGEFNVGSTCHFSCDNG |
|  | H 9 | 2218.6 | RGEFNVGSTCHFSCDNGFKL |
|  | H10 | 2159.5 | FNVGSTCHFSCDNGFKLEGP |
|  | H11 | 2126.4 | GSTCHFSCDNGFKLEGPNNV |
|  | H12 | 2214.4 | CHFSCDNGFKLEGPNNVECT |
|  | H13 | 2072.3 | SCDNGFKLEGPNNVECTTSG |
|  | H14 | 2196.5 | NGFKLEGPNNVECTTSGRWS |
|  | H15 | 2147.4 | KLEGPNNVECTTSGRWSATP |
|  | H16 | 2078.2 | GPNNVECTTSGRWSATPPTC |
|  | H17 | 2108.4 | NVECTTSGRWSATPPTCKGI |
|  | H18 | 2037.5 | CTTSGRWSATPPTCKGIASL |
|  | H19 | 2027.5 | SGRWSATPPTCKGIASLPTP |
|  | H20 | 2025.5 | WSATPPTCKGIASLPTPGLQ |
|  | H21 | 1952.4 | TPPTCKGIASLPTPGLQCPA |
|  | H22 | 1972.5 | TCKGIASLPTPGLQCPALTT |
|  | H23 | 1922.4 | GIASLPTPGLQCPALTTPGQ |
|  | H24 | 1970.4 | SLPTPGLQCPALTTPGQGTM |
|  | H25 | 2095.5 | TPGLQCPALTTPGQGTMYCR |
|  | H26 | 2211.5 | LQCPALTTPGQGTMYCRHHP |
|  | H27 | 2172.5 | PALTTPGQGTMYCRHHPGTF |
|  | H28 | 2209.5 | TTPGQGTMYCRHHPGTFGFN |
|  | H29 | 2215.5 | GQGTMYCRHHPGTFGFNTTC |
|  | H30 | 2340.7 | TMYCRHHPGTFGFNTTCYFG |
|  | I 1 | 2233.5 | CRHHPGTFGFNTTCYFGCNA |
|  | I 2 | 2142.5 | HPGTFGFNTTCYFGCNAGFT |
|  | I 3 | 2134.7 | TFGFNTTCYFGCNAGFTLIG |
|  | I 4 | 2132.6 | FNTTCYFGCNAGFTLIGDST |
|  | I 5 | 2073.6 | TCYFGCNAGFTLIGDSTLSC |
|  | I 6 | 2046.6 | FGCNAGFTLIGDSTLSCRPS |
|  | I 7 | 2110.6 | NAGFTLIGDSTLSCRPSGQW |
|  | I 8 | 2139.6 | FTLIGDSTLSCRPSGQWTAV |
|  | I 9 | 2047.4 | IGDSTLSCRPSGQWTAVTPA |
|  | I10 | 2092.4 | STLSCRPSGQWTAVTPACRA |
|  | I11 | 2121.4 | SCRPSGQWTAVTPACRAVKC |
|  | I12 | 2104.4 | PSGQWTAVTPACRAVKCSEL |
|  | I13 | 2213.4 | QWTAVTPACRAVKCSELHVN |
|  | I14 | 2136.5 | AVTPACRAVKCSELHVNKPI |
|  | I15 | 2181.6 | PACRAVKCSELHVNKPIAMN |
|  | I16 | 2214.6 | RAVKCSELHVNKPIAMNCSN |
|  | I17 | 2244.7 | KCSELHVNKPIAMNCSNLWG |
|  | I18 | 2274.7 | ELHVNKPIAMNCSNLWGNFS |
|  | I19 | 2202.7 | VNKPIAMNCSNLWGNFSYGS |
|  | I20 | 2164.7 | PIAMNCSNLWGNFSYGSICS |
|  | I21 | 2270.7 | MNCSNLWGNFSYGSICSFHC |
|  | I22 | 2221.7 | SNLWGNFSYGSICSFHCLEG |
|  | I23 | 2261.8 | WGNFSYGSICSFHCLEGQLL |
|  | I24 | 2162.7 | FSYGSICSFHCLEGQLLNGS |
|  | I25 | 2065.5 | GSICSFHCLEGQLLNGSAQT |
|  | I26 | 2110.4 | CSFHCLEGQLLNGSAQTACQ |
|  | I27 | 2073.3 | HCLEGQLLNGSAQTACQENG |
|  | I28 | 2130.3 | EGQLLNGSAQTACQENGHWS |
|  | I29 | 2117.3 | LLNGSAQTACQENGHWSTTV |
|  | I30 | 2078.1 | GSAQTACQENGHWSTTVPTC |
|  | J 1 | 2119.1 | QTACQENGHWSTTVPTCQAG |
|  | J 2 | 2130.2 | CQENGHWSTTVPTCQAGPLT |
|  | J 3 | 2140.3 | NGHWSTTVPTCQAGPLTIQE |
|  | J 4 | 2117.4 | WSTTVPTCQAGPLTIQEALT |
|  | J 5 | 2110.5 | TVPTCQAGPLTIQEALTYFG |
|  | J 6 | 2040.5 | TCQAGPLTIQEALTYFGGAV |
|  | J 7 | 1967.5 | AGPLTIQEALTYFGGAVAST |
|  | J 8 | 2025.7 | LTIQEALTYFGGAVASTIGL |
|  | J 9 | 1999.7 | QEALTYFGGAVASTIGLIMG |
|  | J10 | 1942.8 | LTYFGGAVASTIGLIMGGTL |
|  | J11 | 1862.8 | FGGAVASTIGLIMGGTLLAL |
|  | J12 | 1999 | AVASTIGLIMGGTLLALLRK |
|  | J13 | 2217.3 | STIGLIMGGTLLALLRKRFR |
|  | J14 | 2287.3 | GLIMGGTLLALLRKRFRQKD |
|  | J15 | 2304.2 | MGGTLLALLRKRFRQKDDGK |
|  | J16 | 2372.2 | TLLALLRKRFRQKDDGKCPL |
|  | J17 | 2393 | ALLRKRFRQKDDGKCPLNPH |
|  | J18 | 2432.9 | RKRFRQKDDGKCPLNPHSHL |
|  | J19 | 2313.7 | FRQKDDGKCPLNPHSHLGTY |
|  | J20 | 2185.6 | KDDGKCPLNPHSHLGTYGVF |
|  | J21 | 2113.5 | GKCPLNPHSHLGTYGVFTNA |
|  | J22 | 2158.5 | PLNPHSHLGTYGVFTNAAFD |
|  | J23 | 2115.4 | PHSHLGTYGVFTNAAFDPSP |

**S1 Table. Layout of the SPOT Peptide Array of Human P-selectin**
