# Supplementary figures and images for: The vimentin rod domain blocks P-selectin-P-selectin glycoprotein ligand 1 interactions to attenuate leukocyte adhesion to inflamed endothelium
Source: PLoS One. 2020 Oct 13;15(10):e0240164. doi: 10.1371/journal.pone.0240164 (PMC7553327; doi:10.1371/journal.pone.0240164)

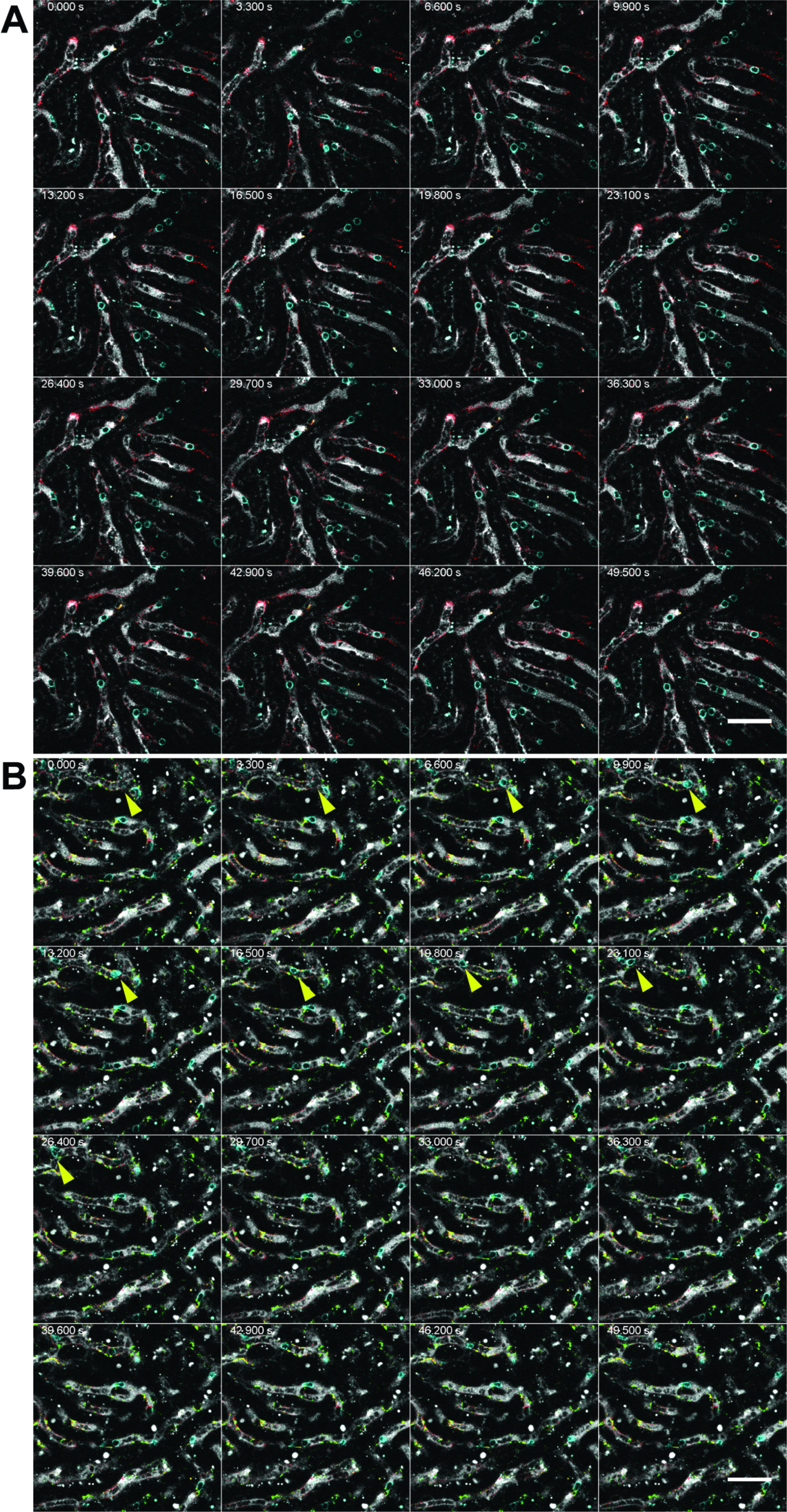

Supplement: S1 Fig — Time-lapsed montage images from intravital microscopy of mouse livers in endotoxemic mice receiving (A) control or (B) Atto 550 labeled rhRod. The benefit of IVM is that it allows for differentiation between firmly adherent and mobile cells. The yellow arrows (B) point to the same PMN (cyan) that is initially immobile, but then quickly flows through the sinusoid (gray) after approximately 1 minute. rhRod (green) tends to colocalize (yellow) with P-selectin (red) along the sinusoids. The 1-minute video can be found in S6 Video (correlating to the montage). Scale bar = 50 μm. (TIF) [file pone.0240164.s003.tif]

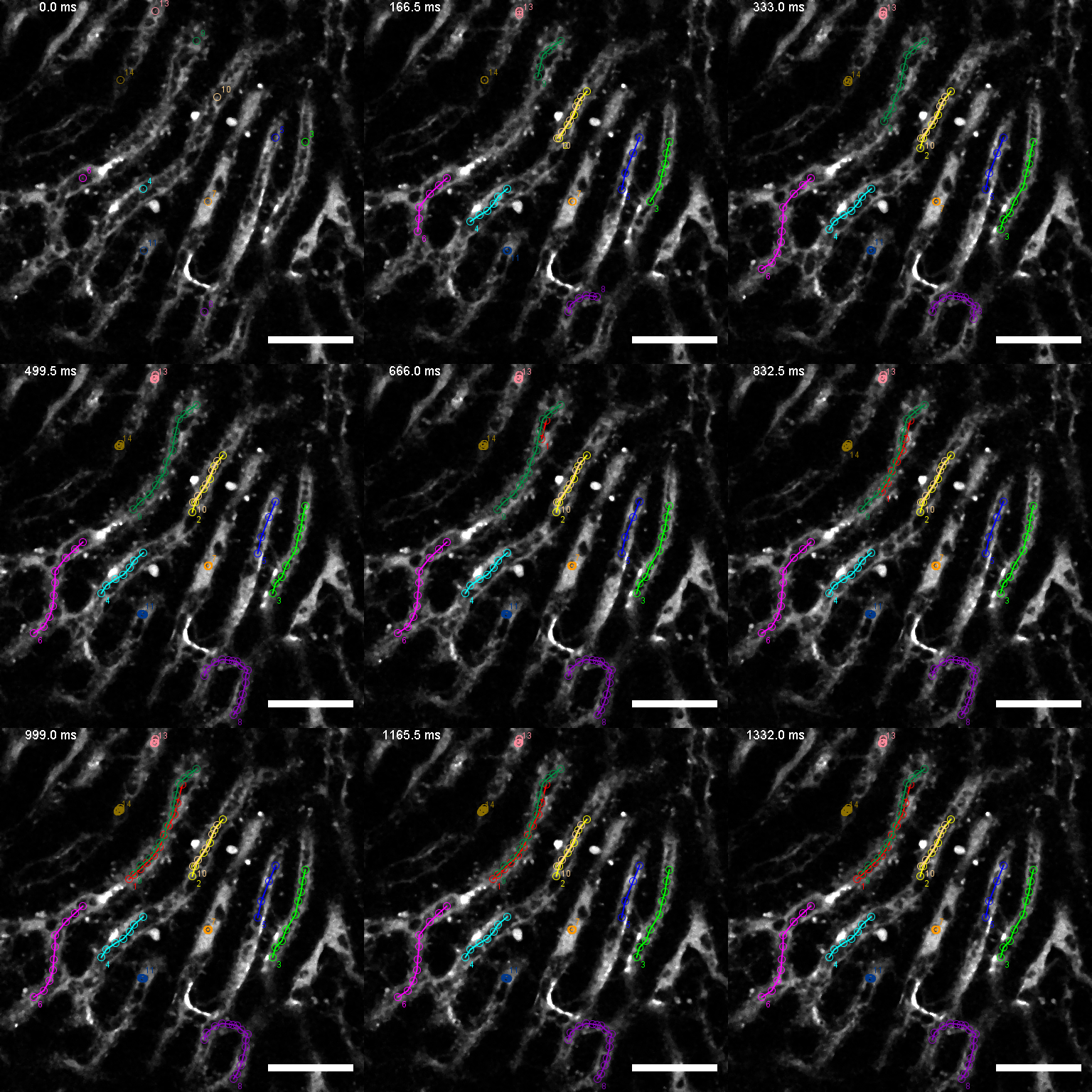

Supplement: S2 Fig — To estimate blood flow velocity in the sinusoids, RBC velocity was used. RBC were identified by negative contrast (black) within the dextran channel (white). The center of RBC were tracked frame by frame (colored lines) and used to calculate the RBC velocity within a segment. Scale bar = 50 μm. (TIF) [file pone.0240164.s004.tif]

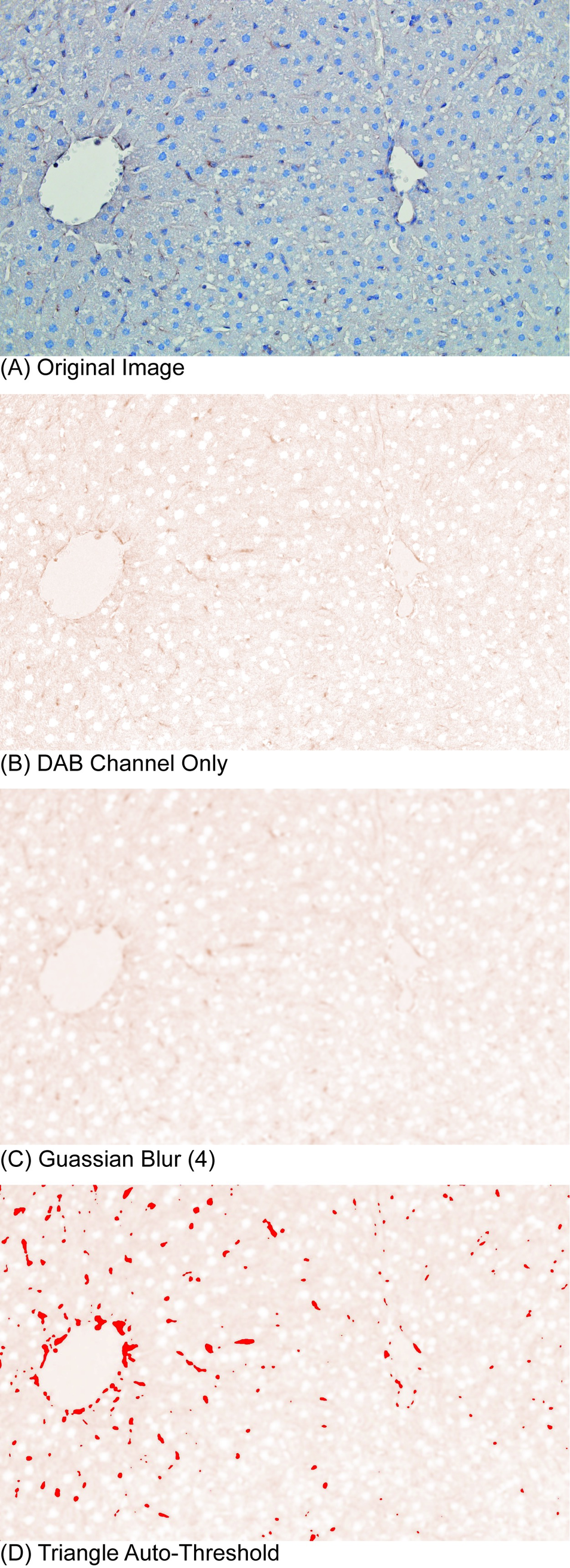

Supplement: S3 Fig — DAB-stained images were processed in FIJI/ImageJ. Each original image with hematoxylin counterstain (A) underwent colour deconvolution using the H DAB vector to isolate the DAB-only channel (B). To reduce background noise, all images underwent a Gaussian filter (radius 4; C). Finally, to determine the area stained by DAB (vimentin positive areas), a triangle auto-threshold was used (D). (TIF) [file pone.0240164.s005.tif]
