# Supplementary material for: The vimentin rod domain blocks P-selectin-P-selectin glycoprotein ligand 1 interactions to attenuate leukocyte adhesion to inflamed endothelium
Source: PLoS One. 2020 Oct 13;15(10):e0240164. doi: 10.1371/journal.pone.0240164 (PMC7553327; doi:10.1371/journal.pone.0240164)
Supplement: S1 Methods — (DOCX) [file pone.0240164.s006.docx]

**S3 Methods. Modelling of rhRod-P-selectin interactions.**

*3TRT-1G1Q Model Parameters for Docking*

HADDOCK clustered 198 structures in 27 clusters, which represents 49.5 % of the water-refined models HADDOCK generated. The cluster with the lowest HADDOCK score is considered the most reliable of all generated structures. The top structure within the lowest scoring cluster was used for analysis.

Cluster 4

| HADDOCK score | -92.0 +/- 10.9 |
| --- | --- |
| Cluster size | 12 |
| RMSD from the overall lowest-energy structure | 1.9 +/- 1.1 |
| Van der Waals energy | -58.2 +/- 8.9 |
| Electrostatic energy | -169.6 +/- 17.1 |
| Desolvation energy | -23.0 +/- 8.2 |
| Restraints violation energy | 230.3 +/- 123.21 |
| Buried Surface Area | 1860.0 +/- 68.2 |
| Z-Score | -2.2 |

*1GK7-1G1Q Model Parameters for Docking*

HADDOCK clustered 121 structures in 18 cluster(s), which represents 30.25 % of the water-refined models HADDOCK generated. The cluster with the lowest HADDOCK score is considered the most reliable of all generated structures. The top structure within the lowest scoring cluster was used for analysis.

Cluster 3

| HADDOCK score | -76.7 +/- 9.9 |
| --- | --- |
| Cluster size | 9 |
| RMSD from the overall lowest-energy structure | 0.6 +/- 0.4 |
| Van der Waals energy | -60.9 +/- 4.2 |
| Electrostatic energy | -68.8 +/- 15.1 |
| Desolvation energy | -22.0 +/- 2.4 |
| Restraints violation energy | 199.4 +/- 117.87 |
| Buried Surface Area | 1751.9 +/- 33.2 |
| Z-Score | -1.6 |
